# A map of copy number variations in the Tunisian population: a valuable tool for medical genomics in North Africa

**DOI:** 10.1038/s41525-020-00166-5

**Published:** 2021-01-08

**Authors:** Lilia Romdhane, Nessrine Mezzi, Hamza Dallali, Olfa Messaoud, Jingxuan Shan, Khalid A. Fakhro, Rym Kefi, Lotfi Chouchane, Sonia Abdelhak

**Affiliations:** 1 Biomedical Genomics and Oncogenetics Laboratory (LR16IPT05), Institut Pasteur de Tunis, Tunis, Tunisia; 2 Department of Biology, Faculty of Science of Bizerte, Jarzouna, Tunisia; 3 Department of Genetic Medicine, Weill Cornell Medicine, New York, NY USA; 4 Department of Microbiology and Immunology, Weill Cornell Medicine, New York, NY USA; 5 Genetic Intelligence Laboratory, Weill Cornell Medicine in Qatar, Education City, Qatar Foundation, Doha, Qatar; 6 Department of Genetic Medicine, Weill Cornell Medical College in Qatar, Doha, Qatar; 7 Department of Human Genetics, Sidra Medicine, Doha, Qatar

**Keywords:** Molecular medicine, Medical genomics

## Abstract

Copy number variation (CNV) is considered as the most frequent type of structural variation in the human genome. Some CNVs can act on human phenotype diversity, encompassing rare Mendelian diseases and genomic disorders. The North African populations remain underrepresented in public genetic databases in terms of single-nucleotide variants as well as for larger genomic mutations. In this study, we present the first CNV map for a North African population using the Affymetrix Genome-Wide SNP (single-nucleotide polymorphism) array 6.0 array genotyping intensity data to call CNVs in 102 Tunisian healthy individuals. Two softwares, PennCNV and Birdsuite, were used to call CNVs in order to provide reliable data. Subsequent bioinformatic analyses were performed to explore their features and patterns. The CNV map of the Tunisian population includes 1083 CNVs spanning 61.443 Mb of the genome. The CNV length ranged from 1.017 kb to 2.074 Mb with an average of 56.734 kb. Deletions represent 57.43% of the identified CNVs, while duplications and the mixed loci are less represented. One hundred and three genes disrupted by CNVs are reported to cause 155 Mendelian diseases/phenotypes. Drug response genes were also reported to be affected by CNVs. Data on genes overlapped by deletions and duplications segments and the sequence properties in and around them also provided insights into the functional and health impacts of CNVs. These findings represent valuable clues to genetic diversity and personalized medicine in the Tunisian population as well as in the ethnically similar populations from North Africa.

## Introduction

Copy number variations (CNVs) are considered as genomic structural variations ranging from 1 kb to multiple megabase pairs in length^1–3^. CNVs are likely caused by one single or a combination of multiple genomic rearrangements, such as unbalanced translocation, deletions, insertions, and duplications. Therefore, CNVs are generally observed as a gain or a loss of DNA segment copies that deviate from the normal diploid state. CNVs may influence phenotypes by changing gene dosage, interrupting coding sequences, creating novel fusion genes, or by altering the distance of a gene from its regulatory elements^4–6^. It has been assessed that up to 60% of the human genome encloses CNVs, which generally range in size from 100 to 500 kb^7^. These CNVs are major contributors to human genetic diversity.

Two models of CNV–phenotype associations have been suggested^8^. The first model encompasses common copy number polymorphisms (CNPs) with a frequency exceeding 1% in the general population. Genes spanned by CNPs are mainly enriched for biological functions and pathways related to drug response, immunity, and sensory perception^9,10^. They alter phenotypes by changing the dosage of genes or other functional elements, thus influencing complex traits such as HIV-1/AIDS susceptibility (MIM 609423), Crohn’s disease (MIM 266600,) and glomerulonephritis in systemic lupus erythematosus (MIM 152700). CNVs also occur in genes encoding drug-metabolizing enzymes, including the cytochrome P450s (*CYP2B6* and *CYP2D6*), which are susceptible to structural variations due to highly homologous pseudogenes. CNV distribution influences drug metabolism and are important in pharmacogenomics screening^11^. The second model involves rare and highly penetrant CNVs. These CNVs are responsible for the deletion or the duplication of large genomic segments resulting in genomic disorders such as Prader–Willi syndrome/Angelman syndrome (MIM 176270/105830, 15q11-q13 deletion), Williams–Beuren syndrome (MIM: 194050, 7q11.23 deletion), Potocki–Lupski syndrome (MIM:610883, 17p11.2 duplication), and Charcot–Marie–Tooth disease, type 1A (MIM:610098, 17p12 duplication)^12^.

In order to understand the extent to which CNVs influence phenotypes, deep analyses in both patient and healthy individuals are required. Different approaches, including quantification of hybridization to specific oligonucleotides^13^, clone arrays^14^, direct genome sequencing^15,16^, and single-nucleotide polymorphism (SNP) array^17–19^, allowed to explore CNVs, thus providing their global estimates of frequencies, distribution, and functional features in large population cohorts and HapMap samples^1,2,4,16,19–29^. Although medical and clinical genetic studies have been widely performed in the Arab World known to display high rates of consanguinity and endogamy, little attention has been paid to potential variations linked to health in the region^30,31^. Therefore, information related to molecular pathogenesis and knowledge of gene variants segregating in the Arab genome is lacking as well as genotype–phenotype correlation of genetic conditions for both monogenic and multifactorial diseases.

Studies focusing on the characterization of CNVs in the Arab World are not available, except one on the Qatari population^16^. In this study, we applied Affymetrix Genome-Wide Human SNP Array 6.0, which was designed for both SNP and CNV detection, to explore genome-wide CNV in the Tunisian population. Tunisia is a North African country with 11 million inhabitants. The native background population is Berber and the genetic properties of the present population are shaped by the multiple invasions and the migratory waves of allogenic populations and ethnic groups mainly from the Middle East and Europe^32^. In addition, like other countries from North Africa and the Middle East, the Tunisian population depicts high rates of consanguinity and endogamy, leading to the expression of recessive genetic diseases at relatively high frequencies and in several cases leading to comorbidity^30,33,34^. Because of the relatively high inbreeding rates in this population^35^, it is likely that CNVs, alongside SNPs and indels, play a role in the inherited disease risk burden. In the present study, we provide the first comprehensive Tunisian CNV map and performed functional analysis on CNV overlapping genes in order to understand their role in conveying disease risk.

## Results

### Characteristics of CNVs identified by PennCNV

A total of 4591 CNV calls on 102 individuals (73 males and 29 females) were merged into adjacent CNVs leading to 4573 CNVs. CNV carrier rate was 75.5%. After filtering unreliable CNV calls from telomeric, centromeric, and immunoglobin regions and removing CNV with <10 probes and according to length, 3964 CNV events were obtained. In this dataset, an average of 38.86 CNV per individual with a ratio of deletions to duplications of about 2:1 was identified (Supplementary Table 1). The number of CNVs per individual ranged from 17 to 63 (Supplementary Fig. 1). The mean size of a CNV was 96.7 kb. The median size of duplications is ~57.170 kb, which is larger than that of deletions (Wilcoxon test, *p* value < 2.2e − 16) (Supplementary Table 1 and Supplementary Fig. 2a, b).

### Characteristics of CNV loci (CNVR) identified by PennCNV

By merging overlapping CNVs into CNV regions (CNVRs), we identified 751 CNVRs with sizes ranging from 1.02 kb to 3.184 Mb and an average size of 104 kb (Supplementary Table 2). Among these CNVR, we identified 469 loci containing only deletions (loss-loci), 173 loci containing only duplications (gain-loci), and 109 loci containing both deletions and duplications (mixed loci). These 751 CNVRs are covering 78.072 Mb of the genome with a sum of loss-loci length of 23.102 Mb, which is slightly larger than that of gain-loci (22.458 Mb). Nevertheless, the CNVR deletion length median (19.380 kb) was significantly lower than that of gain-loci (61.430 kb) (Wilcoxon test *p* value < 2.2e − 16) similarly for the individual CNVs. Moreover, ~40% of CNVR were <20 kb and the majority of these segments were <100 kb (78.5%) (Supplementary Table 3 and Supplementary Fig. 3a). About 60% of the CNVR in this size range (100 kb) were loss-loci, whereas duplication loci represented only 14.24%.

### Characteristics of CNVs identified by Birdseye (Birdsuite)

After filtering spurious CNVs, a total of 6263 segments have been called with an average of ~61.4 CNV per individual (Supplementary Table 1). The count of deletions was nearly three times that of duplications. The number of CNVs per individual ranged from 38 to 325. The mean size of a CNV was 46.770 kb (Supplementary Table 1). The median size of duplication is ~42.770 kb, which is larger than that of deletions (Wilcoxon test, *p* value < 2.2e − 16) (Supplementary Table 1 and Supplementary Fig. 2c, d).

### Characteristics of CNV loci (CNVR) identified by Birdseye (Birdsuite)

We also merged Birdseye output that overlapped into CNVR similarly to the PennCNV output analysis. The 6263 called CNVs were collapsed into 1236 regions, of which 546 were loss-loci, 603 were dup-loci, and the remaining were 87 mixed loci (Supplementary Table 2). The 1236 CNVRs identified by the Birdseye data cover 65.607 Mb of the nucleotide sequence. The sum of the loss-locus lengths (18.971 Mb) is lower than that of duplication loci (29.068 Mb) (Supplementary Table 2). As for the PennCNV data, the median of the loss-locus lengths (13.330 kb) is lower than that of the duplication loci (16.140 kb) in the data generated by Birdseye (Supplementary Table 2). However, this difference is not significant (Wilcoxon test *p* value = 0.8451).

### Comparison of CNVRs generated by both algorithms (PennCNV and Birdsuite)

Significant differences were found when comparing CNVR parameters of data generated by PennCNV and Birdsuite (Supplementary Tables 2 and 3 and Supplementary Fig. 3a, b). The proportion of loss-loci (62.4%) identified by PennCNV data was higher than that for the Birdseye data (44.2%). Nevertheless, the proportions of the duplication (48.8%) were higher in the Birdseye data than that of PennCNV (23%). This difference was significant (*χ*^2^ test *p* value < 2.2e − 16). In addition, the median length of CNVRs generated by PennCNV (28.010 kb) data is twice higher than that of Birdsuite data (16.1 kb) (Wilcoxon test *p* value < 2.2e − 16). In addition, PennCNV tends to call CNVs that collapse into deletion regions longer than those of Birdsuite (Wilcox test *p* value = 3.873e − 06). Similarly, the duplication CNVRs of PennCNV data are longer than that of Birdsuite (Wilcoxon test *p* value < 2.2e − 16). No significant difference was noted for mix-loci lengths between the two algorithms. In addition, CNVR length distributions between the two algorithms were significantly different (Kolmogorov–Smirnov test *p* value = 9.992e − 16). This was also the case for loss-loci and dup-loci lengths (Kolmogorov–Smirnov test *p* value = 4.987e − 05 and *p* value < 2.2e − 16, respectively) (Supplementary Tables 2 and 3).

### Concordance of PennCNV CNVR

As CNV detection using microarrays is usually plagued with poor specificity or a high false-positive rate, and as there is a significant difference between the performance of CNV detection algorithms as shown earlier (Supplementary Tables 1, 2, and 4), we aimed to look for overlapping regions between these two datasets as a concordance and an in silico validation step. Only those detected by both algorithms have been considered. Seventy-eight percent of PennCNV loci overlapped with those of Birdseye (Supplementary Fig. 4a). Consequently, we found 586 loci with 50% reciprocal overlap with Birdseye data on the 22 autosomes called validated CNVR (vCNVR) (Supplementary Fig. 4b). Moreover, deletion and duplication states of PennCNVR output were consistent with Birdseye data in 79% loci. The vCNVR length ranged from 1.02 kb to 2.074 Mb with an average of 90.3 kb. In all, 78.32% of the vCNVR were <100 kb (Supplementary Fig. 5). About 56% of these segments were deletions (del-loci) and 23.2% duplications (dup-loci). Indeed, these 586 vCNVR comprise 102 homozygous deletions (CN = 0), 424 single-copy deletions (CN = 1), 187 single-copy duplications (CN = 3), and 82 amplifications (CN = 4).

The frequencies of these vCNVR genomic segments ranged from relatively uncommon (0.98%) to polymorphic (98%) (Supplementary Table 5). About 56% of these validated CNVRs were singleton loci, thus reported only in one individual (0.98%). Therefore, 259 vCNVR (44.2%) were polymorphic (frequency ≥ 1%). Among these, 150 (25.6%) were exclusively deletions (CN = 0 or CN = 1) and 21 (3.6%) exclusively duplications (CN = 3 or CN = 4). Eighty-eight (15.02 %) were reported as “mixed” loci.

### Characteristics of CNPs identified by Canary (Birdsuite)

In addition to the identification of CNV segments by PennCNV and Birdseye, we also genotyped previously reported CNPs. Among the 1291 autosomal CNPs, 683 (52.9%) were allelic in the Tunisian population (meaning being deletions and/or duplications) and 530 (41.05%) were bi-allelic. CNPs were genotyped on all the autosomes (Supplementary Fig. 6). The CNP frequencies ranged from relatively uncommon (0.98%) to polymorphic (99%) (Supplementary Table 5). Therefore, 650 (95.2%) of these allelic CNPs were polymorphic (allelic frequency > 1%).

The CNP length ranged from 1.017 to 487.878 kb with an average of 22.827 kb. In all, 94.87% of the genotype CNPs are <100 kb (Supplementary Fig. 7). About 61.05% of these segments were exclusively deletions (417 del-loci) and 16.54% exclusively duplications (113 dup-loci). The count of the CNP loci deletions was therefore near 3.69 times that of duplications (Supplementary Table 6). Similarly, the gain CNP loci are longer than loss CNP loci (Wilcoxon test *p* value = 3.8 × 10^−08^). In addition, CNP length distributions between the two types of loci were significantly different (Kolmogorov–Smirnov test *p* value = 4.43 × 10^−07^).

These 683 allelic CNP comprise 235 homozygous deletions (CN = 0), 559 single-copy deletions (CN = 1), 223 single-copy duplications (CN = 3), 92 amplifications (CN = 4), 5 amplifications (CN = 5), and 4 amplifications (CN = 6). About 96.78% (661) of these CNPs were non-singleton loci, thus polymorphic (frequency ≥ 1%).

### Characteristics of the global CNV map

**Fig. 1:**
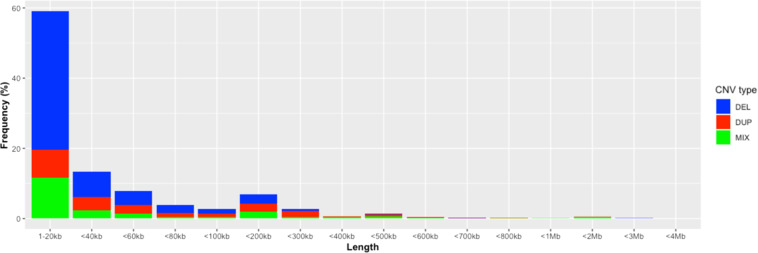
Global map CNV size distribution. Overall length distribution of the global CNV map.

As vCNVR and CNP overlapped (169 overlapping vCNVRs), we merged them by union together and checked them manually in order to provide a global CNV map of the Tunisian population. After removing likely false-positive segments with frequency >90% that were absent from the 1000 Genomes project and Genome Aggregation database (gnomAD) databases, the global CNV map is composed of 1083 CNVs (Supplementary Fig. 8 and Supplementary Dataset). The deletions represent 57.43% of the identified CNVs, while the duplications and the mixed loci are less represented (21.79% and 20.77%, respectively) (Supplementary Fig. 8 and Supplementary Dataset). The CNV length ranged from 1.017 Kb to 2.074 Mb with an average of 56.734 kb. The genome coverage has been evaluated to 61.443 Mb. The overall length distribution of the global CNV map showed that most of them (80.24%) were small in length (60 kb) (Fig. 1). The lengths of amplifications (median = 29.943 kb) were significantly greater than those of deletions (median = 10.084 kb) (Wilcoxon test *p* value < 2.2 × 10^–16^) (Supplementary Table 7). Homozygous loss segments represent 25.3% of the identified CNVs and are shorter than heterozygous deletions (median 8.904 vs. 10.490 kb, Wilcoxon test *p* value = 0.04). Approximately 28% (27.97%) of all the reported CNVs were singleton segments, meaning, identified in one individual and therefore having a frequency <1%.

**Fig. 2:**
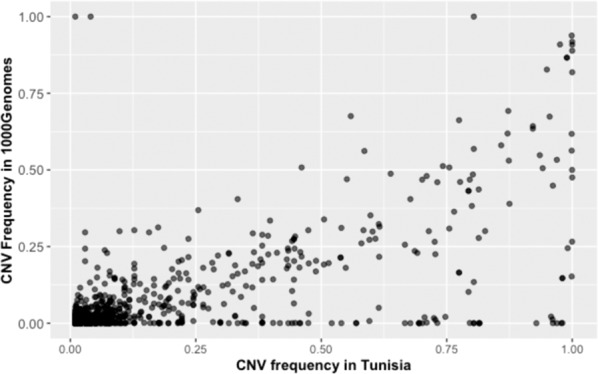
Scatter plot of frequencies of CNVs in Tunisia identified in the 1000 Genome project. A positive and high correlation is shown (Pearson correlation *r* = 0.70, 95% CI = [0.67;0.73], *p* value < 2.2e – 16).

The pairwise correlation between the CNV frequency of our dataset overlapping with those of the 1000 Genomes was positive, high, and significant (Pearson correlation *r* = 0.70, 95% confidence interval (CI) = [0.67;0.73], *p* value < 2.2e − 16) (Fig. 2). The pairwise correlation was also positive, high, and significant at the population level among the five continental groups of the 1000 Genome project: Africa (AFR): *r* = 0.6, 95% CI = [0.57,0.63], *p* value < 2.2e − 16; the America (AMR): *r* = 0.69, 95% CI = [0.67,0.72], *p* value < 2.2e − 16; Europe (EUR): *r* = 0.72, 95% CI = [0.69,0.74], *p* value < 2.2e − 16; East Asia (EAS): *r* = 0.63, 95% CI = [0.6, 0.66], *p* value < 2.2e − 16; South Asia (SAS): *r* = 0.65, 95% CI = [0.64,0.70], *p* value < 2.2e − 16 (Supplementary Fig. 9a–e). These results suggest that the CNV profile of the Tunisian population is similar to the European population and different from the African.

In addition, in order to identify novel CNVs, we queried the following databases: Database of Genomic Variants (DGV) (http://dgv.tcag.ca/dgv/app/home), 1000 Genomes, dbVar (https://www.ncbi.nlm.nih.gov/dbvar/), and gnomAD, as well as the Deciphering Developmental Disorders (DDD) and Ira M. Hall’s lab studies for 50% overlapping segments. Seven “novel” CNVs were found, of which one is a deletion and the remaining are duplications (Table 1). All these “novel” segments are singletons. The length of these “novel” CNVs ranges from 7.155 to 700.721 kb with an average of 250.699 kb (Table 1).

**Table 1:** Novel CNVs identified in the Tunisian population.

CNV ID	Chr:start–end	CNV length (kb)	CNV type	Frequency (%)	RefSeq gene	OMIM phenotype
CNVR_2_19	2:66,077,241–66,249,560	172.319	DEL	0.98		
CNVR_4_8	4:38,841,353–39,120,619	279.266	DUP	0.98	*FAM114A1; KLHL5; MIR574; TLR6; TMEM156*	No
CNVR_11_27	11:111,478,291–111,677,695	199.404	DUP	0.98	***ALG9***; ***PPP2R1B***; *SIK2*	Yes
CNVR_11_29	11:128,374,712–128,381,867	7.155	DUP	0.98	*ETS1*	No
CNVR_13_8	13:61,040,537–61,741,258	700.721	DUP	0.98	*LINC00378; TDRD3*	No
CNVR_13_15	13:112,804,834–113,081,675	276.841	DUP	0.98	*LINC01043; LINC01044; LINC01070; LOC100506016; LOC101928730; SPACA7*	No
CNVR_14_11	14:50399836–50519022	119.186	DUP	0.98	*LINC01588; LINC01599; MIR6076*	No

Gene in bold indicates genes involved in OMIM phenotype.

### Functional effect of CNV overlapping genes and pathway enrichment

**Fig. 3:**
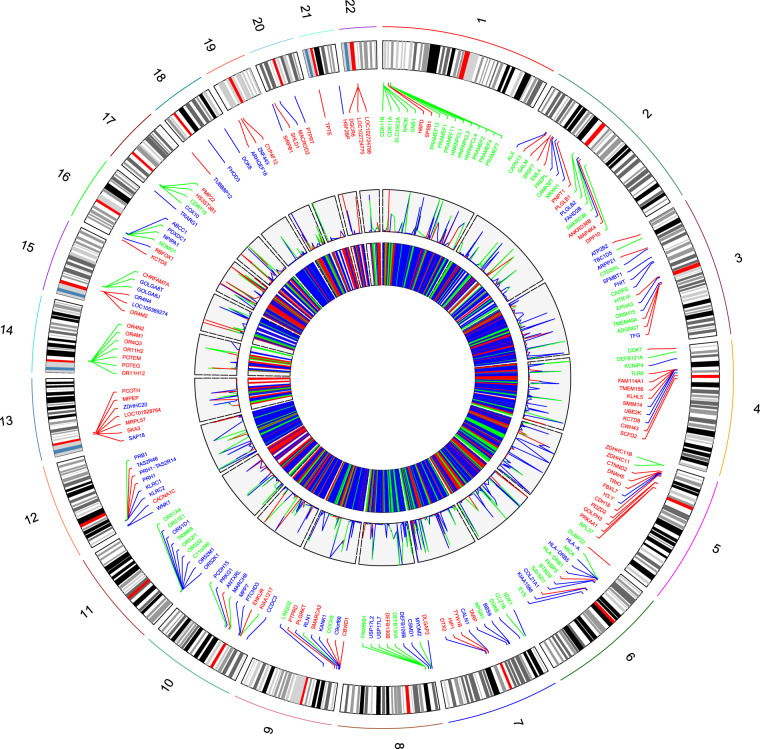
Circular plot showing a chromosomal view of the global CNV map of the Tunisian population. The innermost circle with vertical lines represents all the CNV from chromosomes 1 to 22: blue, red, and green color lines represent deletions, duplications, and mixed loci, respectively. The frequency of each CNV is depicted by the second track. The third concentric circle represents the genomic distribution of CNV genes overlapped according to the type of the CNV.

**Fig. 4:**
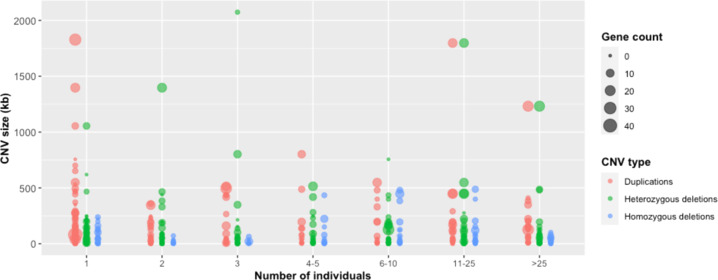
CNV length, gene content and frequency distributions. CNVs were plotted according to event type (color), size (*y*-axis), frequency in the Tunisian population (*x*-axis, number of individuals), and number of RefSeq genes affected (circle size).

The 524/1083 (48.38 %) CNVs overlap with 1018 RefSeq genes (597 protein coding genes, 421 non-coding genes) (Fig. 3 and Supplementary Dataset). Deletions spanning genes were more frequent (261 genic deletions vs. 151 genic duplications) (Table 2, Supplementary Table 8, and Supplementary Dataset). Homozygous deletions are significantly gene poor (Kruskal–Wallis *χ*^2^ = 37.202, d.f. = 2, *p* value = 8.348e − 09) (Fig. 4). Rare CNVs (frequency < 1%) seem not to harbor more genes than common events (Wilcoxon rank-sum test *p* value = 0.1972). The longest CNV in our dataset is a heterozygous deletion at the long intergenic non-protein coding RNA 290 (LINC00290) locus reported in three different healthy individuals.

**Table 2:** Functional annotation of CNVs in the Tunisian population.

	Total	Non-genic	Genic	Protein coding genic	Coding genes	Non-coding protein genic	Non-coding genes	miRNA	Genic CNV affecting Mendelian disease genes	Mendelian genes affected	dbVar (*)	1000 Genomes	DGV	gnomAD	DDD	IMH	Promoter site
CN = 0- homozygous deletions	274	157	117	88	120	50	71	3	19	18	4	229	120	250	100	149	58
CN = 1- heterozygous deletions	830	465	365	283	399	146	257	35	70	66	11	667	204	741	266	484	144
CN = 3 - duplication	314	133	181	178	381	108	258	42	46	59	11	219	93	264	119	178	113
CN = 4-amplification	161	65	96	72	163	52	180	25	15	18	4	109	82	136	71	64	63
CN = 5-amplification	3	0	3	3	10	3	6	0	1	4	0	2	3	2	2	1	3
CN = 6-amplification	2	0	2	2	13	1	2	0	1	4	0	1	2	1	2	1	2

(*) = Status (pathogenic and likely pathogenic).

Among the novel CNVs, 6 are overlapping with 20 RefSeq genes (10 protein coding genes and 10 non-protein coding genes) (Table 1).

Pathway analysis of these genes revealed 16 significant enriched pathways of potential concern for public health (Table 3). Furthermore, standard functional annotation using the GO terms and pathways showed significant cellular component terms relating to the nervous system (Supplementary Table 9). Biological processes and molecular functions of genes bearing CNVs were also related to physiology of the nervous system, drug metabolism, carbohydrate metabolism, immunological system, cardiac and lung organs, and olfactory and auditory systems (Supplementary Table 9).

**Table 3:** KEGG pathways enriched in genes affected by CNVs in the Tunisian population.

KEGG pathway	Number of genes	Fold enrichment	*P* value
Olfactory transduction	37	3.16	7.66 × 10^−10^
Chemical carcinogenesis	15	6.38	5.81 × 10^−8^
Drug metabolism—cytochrome P450	14	7.01	5.86 × 10^−8^
Metabolism of xenobiotics by cytochrome P450	14	6.44	1.67 × 10^−7^
Drug metabolism—other enzymes	7	5.18	0.002
Starch and sucrose metabolism	6	6.19	0.002
Retinol metabolism	8	4.26	0.002
Glutathione metabolism	7	4.67	0.003
Antigen processing and presentation	8	3.58	0.006
Carbohydrate digestion and absorption	6	4.86	0.007
Steroid hormone biosynthesis	6	3.52	0.026
Biosynthesis of unsaturated fatty acids	4	5.92	0.028
Porphyrin and chlorophyll metabolism	5	4.05	0.033
Osteoclast differentiation	9	2.34	0.037
Mineral absorption	5	3.87	0.039
Ascorbate and aldarate metabolism	4	5.04	0.043

### Mapping CNV genes to diseases and phenotypes

In order to determine whether CNV might play a role in disease expression in the Tunisian population, we queried the 597 RefSeq genes affected by the CNV segments against the GAD database^36^, which is an archive of human genetic association studies of complex diseases. We found genes associated with 255 diseases clustered in 10 disease classes in this database (Supplementary Table 10). Significant disease classes include diseases of the immune and blood systems and metabolic diseases (Supplementary Table 10). Diseases of the nervous system are also present. CNV genes seems also to underlie or to be associated with aging and ocular disorders.

**Fig. 5:**
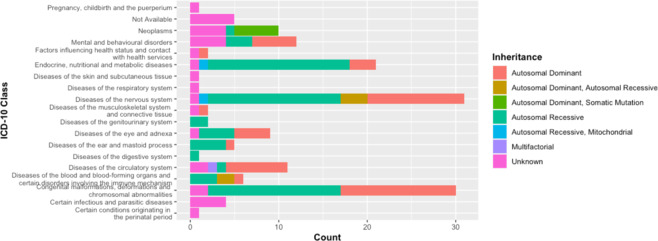
WHO ICD-10 classification of diseases caused by CNV genes. Three major disease groups are caused by CNV genes. Disease classes are colored according to the inheritance mode.

**Fig. 6:**
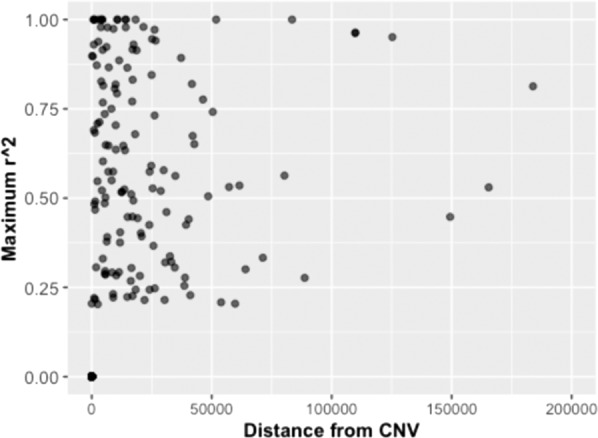
The correlation between the *r*^2^ and the distance between CNV deletion and single-nucleotide polymorphism (SNP). The decrease of the *r*^2^ strength as the distance of the CNVs and the SNP increases is shown.

In addition, these RefSeq genes were compared to the database of Online Mendelian Inheritance in Man (OMIM). One hundred and three genes were reported to cause 155 Mendelian diseases/phenotypes (Supplementary Dataset). According to the World Health Organization international classification of diseases (WHO ICD), genic CNVs are responsible for three major disease groups: (1) diseases of the nervous system (20%), (2) the congenital malformations, deformations, and chromosomal abnormalities (19.35%), and (3) endocrine, nutritional, and metabolic diseases (13.55%). Mental and behavioral disorders as well as neoplasm represented 7.74% and 6.45% of the diseases, respectively (Fig. 6). Among the CNV predisposing diseases, 41.93% are autosomal recessive (AR) and 30.32% autosomal dominant (AD) (Fig. 5).

In order to identify genes that have evidence of disease, we performed a manual annotation of CNV-overlapped genes listed in OMIM to determine the number of exons and introns that were potentially disrupted by these CNVs. Consequently, we focused on functional deletions that affect exons as well as functional duplications that either overlap at least one entire gene, thus likely leading to an increased dosage, or those that are internal to the gene that potentially could disrupt the protein Heterozygous deletions leading to the loss of genomitranslation frame. In addition, we discarded all intronic events. Sixty-three (63) disease genes were identified as harboring 54 CNVs (Table 4). The list of OMIM disease genes was then split into two groups. The first one includes CNVs that have been previously reported in the 1000 Genome project, gnomAD, the DGV, and dbVar databases, as well as those identified in the studies of DDD and Ira’s lab. The second one contains disease genes affected by the novel, likely Tunisian-specific CNV, that were not reported by the mentioned queried databases.

**Table 4:** Known and novel CNVs in the Tunisian population affecting OMIM Mendelian disease genes.

Phenotypes	OMIM phenotype	Inheritance	RefSeq gene symbol	CNV location	CNV ID	CNV type	Chr:start–end	CNV length (kb)	CNV category	CNV frequency (%)
Somatic acute lymphoblastic leukemia; mental retardation 42	613065; 616973	Unk; AD	*GNB1*	Intron5-txEnd	CNVR_1_1	MIX	1:1,627,917–1,730,086	102.169	Known	4.90
Cortisone reductase deficiency 1	604931	AR	*H6PD*	Intron3-txEnd	CNVR_1_2	DUP	1:9,315,846–9,402,403	86.557	Known	0.98
Uncombable hair syndrome	191480	AR	*PADI3*	Intron6-txEnd	CNVR_1_5	DUP	1:17,594,895–17,620,009	25.114	Known	0.98
Rh-negative blood type	Not Available	Unk	*RHD*	txStart-txEnd	CNVR_1_9_CNP28	MIX	1:25,593,128–25,663,344	70.216	Known	43.14
Dihydropyrimidine dehydrogenase deficiency; 5-fluorouracil toxicity	274270; 274270	AR	*DPYD*	Intron16-intron18	CNVR_1_18	DEL	1 :97,747,723–97,798,478	50.755	Known	0.98
Fontaine progeroid syndrome	612289	AD	*SLC25A24*	txStart-intron1	CNP84	MIX	1 :108,734,235–108,737,496	3.261	Known	17.5
Familial atrial fibrillation 11; digenic atrial standstill (GJA5/SCN5A)	614049; 108770	AD; AD	*GJA5*	txStart-txEnd	CNVR_1_27	DUP	1:146,101,239–147,929,336	1828.097	Known	0.98
Multiple types cataract 1	116200	AD	*GJA8*	txStart-txEnd	CNVR_1_27	DUP	1:146,101,239–147,929,336	1828.097	Known	0.98
Resistance to malaria; susceptibility to systemic lupus erythematosus	611162; 152700	Unk; AD	*FCGR2B*	txStart-txEnd	CNVR_1_31_CNP118	MIX	1:161,517,938–161,660,040	142.102	Known	96.08
Autoimmune thrombocytopenic purpura	188030	AD	*FCGR2C*	txStart-txEnd	CNVR_1_31_CNP118	MIX	1:161,517,938–161,660,040	142.102	Known	96.08
Immunodeficiency 20	615707	AR	*FCGR3A*	txStart-intron3	CNVR_1_31_CNP118	MIX	1:161,517,938–161,660,040	142.102	Known	96.08
Alloimmune neonatal neutropenia	Not Available	Unk	*FCGR3B*	txStart-txEnd	CNVR_1_31_CNP118	MIX	1:161,517,938–161,660,040	142.102	Known	96.08
Basal laminar drusen; complement factor H deficiency; susceptibility to atypical hemolytic uremic syndrome 1; sge-related macular degeneration 4	126700; 609814; 235400; 610698	AD; AR, AD; AR, AD; Unk	*CFH*	Intron 15-txEnd	CNVR_1_37	MIX	1:196,705,045–196,909,645	204.600	Known	70.59
Susceptibility to atypical hemolytic uremic syndrome; reduced risk of age-related macular degeneration	235400; 603075	AR, AD; AD	*CFHR1*	txStart-txEnd	CNVR_1_37	MIX	1:196,705,045–196,909,645	204.600	Known	70.59
Susceptibility to atypical hemolytic uremic syndrome; reduced risk of age-related macular degeneration	235400; 603075	AR, AD; AD	*CFHR3*	txStart-txEnd	CNVR_1_37	MIX	1:196,705,045–196,909,645	204.600	Known	70.59
Susceptibility to atypical hemolytic uremic syndrome; reduced risk of age-related macular degeneration	235400; 603075	AR, AD; AD	*CFHR1*	txStart-txEnd	CNP147	DEL	1:196,731,035–19,680,2072	71.037	Known	58
Susceptibility to atypical hemolytic uremic syndrome; reduced risk of age-related macular degeneration	235400; 603075	AR, AD; AD	*CFHR3*	txStart-txEnd	CNP147	DEL	1:196,731,035–196,802,072	71.037	Known	58
Premature ovarian failure 12; spermatogenic failure 15	616947; 616950	AR; AR	*SYCE1*	Intron1-txEnd	CNP1670	DUP	10:135,328,663–135,377,278	48.615	Known	14
Blood group, Indian system	609027	Unk	*CD44*	txStart-intron1	CNVR_11_13	DEL	11:35,147,497–35,161,076	13.579	Known	0.98
Congenital disorder of glycosylation type Il; Gillessen–Kaesbach–Nishimura syndrome	608776; 263210	AR; AR	*ALG9*	Intron14-txEnd	CNVR_11_27	DUP	11:111,478,291–111,677,695	199.404	Novel	0.98
Lung cancer	211980	AR	*PPP2R1B*	txStart-txEnd	CNVR_11_27	DUP	11:111,478,291–111,677,695	199.404	Novel	0.98
Nephronophthisis 15	614845	AR	*CEP164*	Exon32-txEnd	CNVR_11_28	DUP	11:117,282,799–117,350,336	67.537	Known	0.98
Spermatogenic failure 9	613958	AR	*DPY19L2*	txStart-intron 21	CNVR_12_14_CNP12002	MIX	12:63,961,449–64,120,642	159.193	Known	1.96
Combined oxidative phosphorylation deficiency 31	617228	AR	*MIPEP*	txStart-intron10	CNVR_13_2	DUP	13:24,429,453–24,656,151	226.698	Known	0.98
Combined oxidative phosphorylation deficiency 27	616672	AR	*CARS2*	txStart-txEnd	CNVR_13_14	DUP	13:111,047,100–111,596,127	549.027	Known	0.98
Susceptibility to intracerebral hemorrhage; porencephaly 2	614519; 614483	Unk;AD	*COL4A2*	Intron4-txEnd	CNVR_13_14	DUP	13:111,047,100–111,596,127	549.027	Known	0.98
Somatic head and neck squamous cell carcinoma	275355	Unk	*ING1*	txStart-txEnd	CNVR_13_14	DUP	13:111,047,100–111,596,127	549.027	Known	0.98
Hereditary sensory neuropathy type ID; spastic paraplegia 3A	613708; 182600	AD; AD	*ATL1*	Intron5-txEnd	CNVR_14_12	DUP	14:51,060,651–51,148,978	88.327	Known	0.98
Molybdenum cofactor deficiency C	615501	AR	*GPHN*	Intron8-txEnd	CNVR_14_13	DUP	14:67,456,832–67,688,528	231.696	Known	0.98
Neurophysiologic defect in schizophrenia	Not Available	Unk	*CHRNA7*	Intron9-txEnd	CNVR_15_8	MIX	15:32,458,660–32,876,984	418.324	Known	6.86
Deafness 16	603720	AR	*STRC*	Intron23-exon26	CNP12319	MIX	15:43,892,841–43,894,806	1.965	Known	3.61
Altered CES1-related drug metabolism	618057	Unk	*CES1*	Intron3-txEnd	CNVR_16_11	DUP	16:55,829,556–55,858,793	29.237	Known	2.94
Primary ciliary dyskinesia 5	608647	AR	*HYDIN*	Intron17-intron83	CNVR_16_16	DUP	16:70,854,380–71,095,320	240.940	Known	0.98
Joubert syndrome 20; Meckel syndrome 11	614970; 615397	AR; AR	*TMEM231*	Intron5-txEnd	CNVR_16_17_CNP2200	DUP	16:75,558,083–75,575,999	17.916	Known	1.96
Microcornea–myopic chorioretinal atrophy–telecanthus	615458	AR	*ADAMTS18*	txStart-txEnd	CNVR_16_18	MIX	16:76,884,555–77,939,493	1054.938	Known	1.96
Hypercarotenemia and vitamin A deficiency	115300	AD	*BCO1*	txStart-txEnd	CNVR_16_22	DUP	16:81,200,628–81,347,435	146.807	Known	0.98
Mental retardation 45	615979	AR	*FBXO31*	Intron6-txEnd	CNVR_16_23	DUP	16:87,337,542–87,369,578	32.036	Known	1.96
Leigh syndrome due to mitochondrial COX4 deficiency; mitochondrial complex IV deficiency	256000; 220110	AR, Mito; AR, Mito	*COX10*	Intron5-txEnd	CNVR_17_4	MIX	17:14,094,261–15,491,545	1397.284	Known	2.94
Charcot–Marie–Tooth disease type 1A; Charcot–Marie–Tooth disease type 1E; Dejerine–Sottas disease; inflammatory demyelinating neuropathy; recurrent neuropathy with pressure palsies; Roussy–Levy syndrome	118220; 118300; 145900; 139393; 162500; 180800	AD; AD; AR, AD; AD; AD; AD	*PMP22*	txStart-txEnd	CNVR_17_4	MIX	17:14,094,261–15,491,545	1397.284	Known	2.94
Susceptibility to HIV/AIDS	609423	Unk	*CCL3L1*	txStart-txEnd	CNVR_17_9	DUP	17:34,436,266–34,629,696	193.430	Known	17.65
Koolen–De Vries syndrome	610443	AD	*KANSL1*	txStart-intron4	CNP2269	DUP	17:44,165,801–44,364,214	198.413	Known	35.29
Coumarin resistance; resistance to lung cancer; protection from nicotine addiction	122700; 211980; 188890	AD; AR; Unk	*CYP2A6*	txStart-intron7	CNVR_19_7_CNP2415	DEL	19:41,350,995–41,380,946	29.951	Known	2.94
Spastic paraplegia 73	616282	AD	*CPT1C*	Intron12-txEnd	CNVR_19_9	DUP	19:50,210,715–50,240,557	29.842	Known	0.98
Delayed/rapid progression to AIDS	609423	Unk	*KIR3DL1*	txStart-txEnd	CNVR_19_15	MIX	19:55283842–55,373,818	89.976	Known	6.86
Congenital myasthenic syndrome 22	616224	AR	*PREPL*	txStart-intron8	CNVR_2_13	DEL	2:44,564,517–44,631,600	67.083	Known	0.98
Combined oxidative phosphorylation deficiency 13; deafness AR 70	614932; 614934	AR; AR	*PNPT1*	txStart-exon2	CNP225	DUP	2:55,914,790–55,938,579	23.789	Known	1.98
Hyperprolinemia type I; susceptibility to schizophrenia 4	Not Available	AR; AD	*PRODH*	txStart-txEnd	CNVR_22_1_CNP12789	DUP	22:18,810,110–19,006,125	196.015	Known	4.90
Modifier of deafness 12	601386	AR	*ATP2B2*	Intron2-intron5	CNVR_3_7	DUP	3:10,438,324–10,454,893	16.569	Known	0.98
Hereditary motor and sensory neuropathy, Okinawa type; spastic paraplegia 57	604484; 615658	AD; AR	*TFG*	txStart-intron3	CNVR_3_21	DUP	3:100,294,518–100,442,509	147.991	Known	1.96
Propionicacidemia	606054	AR	*PCCB*	txStart-txEnd	CNVR_3_28	DUP	3:135,889,795–136,332,216	442.421	Known	0.98
Mental retardation 47	617635	AD	*STAG1*	Intron3-txEnd	CNVR_3_28	DUP	3:135,889,795–136,332,216	442.421	Known	0.98
Bone mineral density QTL 12, osteoporosis	612560	Unk	*UGT2B17*	txStart-txEnd	CNVR_4_11	MIX	4:69,198,403–69,582,483	384.080	Known	39.21
Bone mineral density QTL 12, osteoporosis	612560	Unk	*UGT2B17*	txStart-txEnd	CNP603	DEL	4:69,360,488–69,485,979	125.491	Known	55.1
Hypogonadotropic hypogonadism 11 with or without anosmia	614840	AR	*TACR3*	Intron1-txEnd	CNVR_4_19	DUP	4:104,487,285–104,598,947	111.662	Known	0.98
Blood group, Ss; resistance to malaria	611162	Unk;Unk	*GYPB*	txStart-txEnd	CNVR_4_33	DEL	4:144,842,637–144,943,609	100.972	Known	0.98
Blood group, Ss; resistance to malaria	611162	Unk;Unk	*GYPB*	Intron1-exon3	CNP10807	DEL	4:144,920,574–144,924,937	4.363	Known	1.09
Primary ciliary dyskinesia 3 with or without situs inversus	608644	Unk	*DNAH5*	txStart-intron47	CNVR_5_8	DUP	5:13,798,818–14,170,256	371.438	Known	0.98
Mental retardation 44	617061	AD	*TRIO*	txStart-intron1	CNVR_5_8	DUP	5:13,798,818–14,170,256	371.438	Known	0.98
Molybdenum cofactor deficiency B	252160	AR	*MOCS2*	txStart-intron1	CNP799	DEL	5:52,404,519–52,409,439	4.920	Known	58.14
Susceptibility to carbamazepine-induced hypersensitivity syndrome	608579	Unk	*HLA-A*	txStart-txEnd	CNVR_6_4_CNP928	MIX	6:29,843,433–29,921,127	77.694	Known	100
Major depressive disorder and accelerated response to antidepressant drug treatment	608516	Unk	*FKBP5*	Intron6-txEnd	CNP11052	DUP	6:35,505,311–35,564,811	59.500	Known	1.05
Barrett esophagus/esophageal adenocarcinoma	614266	Unk	*MSR1*	Intron5-txEnd	CNVR_8_14	DEL	8:15,949,976–16,021,468	71.492	Known	1.96
Mental retardation 13	613192	AR	*TRAPPC9*	txStart-intron16	CNVR_8_40	DUP	8:141,257,195–141,554,463	297.268	Known	0.98
Hyper-IgE recurrent infection syndrome	243700	AR	*DOCK8*	txStart-intron2	CNP11533	DUP	9:149,481–274,606	125.125	Known	2.02
Early infantile epileptic encephalopathy, 37	616981	AR	*FRRS1L*	Intron2-txEnd	CNVR_9_27	DEL	9:111,883,683–111,909,995	26.312	Known	0.98
Maturity-onset diabetes of the young type VIII	609812	AD	*CEL*	Intron7-txEnd	CNP11660	DEL	9:135,943,214–135,957,440	14.226	Known	1.03
Blood group, ABO system	616093	Unk	*ABO*	Exon 6-exon 7	CNP11661	DEL	9:136,128,542–136,132,873	4.331	Known	1.27

*AD* autosomal dominant, *AR* autosomal recessive, *Mito* mitochondrial, *Unk* unknown, *txStart* transcript start, *txEnd* transcript end.

In the former group, we reported 56 genes affected by 53 known CNV segments (13 deletions, 27 duplications, and 13 mixed loci) (Table 4). Frequency of these segments range from 0.98 to 100% (Table 4). Among these CNV known segments, heterozygous deletions in two individuals have been detected spanning from exons 6 to 7 in the *ABO* gene. Three exons have been deleted at the heterozygous state in the *STRC* gene known to cause deafness. Two exons are also deleted at the heterozygous state in the *COX10* gene responsible for the Leigh syndrome. Heterozygous deletions leading to the loss of genomic segment spanning from the start of the transcript to intron 21 in the spermatogenic failure disorder gene have also been reported. As these disorders are AR and the individuals harbor these deletions at the heterozygous state, consequently, they do not express the corresponding phenotypes.

In addition, homozygous deletions (in 13% of the sample) and heterozygous deletions (in 23%) overlapping from intron 15 to the end of the *CFH* gene known to cause three diseases have been identified. These diseases are: basal laminar drusen (AD), complement factor H deficiency (AR, AD), and atypical hemolytic uremic syndrome (AR, AD). Unfortunately, no additional phenotype data are available to check if these CNVs were causative of one of these phenotypes in the genotyped individuals of our cohort. Moreover, 16 CNVs completely delete 18 genes including *RHD*. Complete gene deletions affect, for example, the *CFHR1*, *CFHR3*, and *PMP22* genes known to predispose to hemolytic uremic syndrome and to cause Charcot–Marie–Tooth disease type 1A, Charcot–Marie–Tooth disease type 1E, Dejerine–Sottas disease, and Roussy–Levy syndrome (Table 4).

Heterozygous deletion leading to the disruption of the coding sequence of the *FRRS1L* gene has been identified. This gene is the cause of the early infantile epileptic encephalopathy, an AR disease. Complete gene duplication in AR genes like *CARS2* responsible for combined oxidative phosphorylation deficiency has been observed (Table 4).

We also examined OMIM-gene-containing likely novel CNVs. In this group, we identified one singleton duplication affecting the *ALG9* gene and the complete *PPP2R1B* gene (Table 4). The two genes are known to be the causes of the congenital disorder of glycosylation type II and lung cancer, respectively (Table 4).

### Linkage disequilibrium (LD) analysis of CNV with nearby SNP

We investigated the “taggability” (measuring the CNVs that are in high LD with flanking SNPs) of SNPs to CNVs in the Tunisian population. We restricted our LD analysis to polymorphic bi-allelic deletions identified in at least four individuals (frequency ≥ 3.9%). Therefore, 223 bi-allelic deletions (bi-deletions) were retained for this analysis (Supplementary Dataset). The LD analysis between bi-deletions and flanking SNP was performed within five windows (200 kb, 500 kb, 1 Mb, 2 Mb, and 3 Mb). As expected, all the bi-deletions had at least one neighboring SNP within the genomic region of either breakpoint. The maximum number and the median of SNPs in LD with CNVs were 15 and 4, respectively. This result indicates that half the bi-allelic deletions could be tagged by >4 SNPs and that some of these deletions could be tagged by 15 SNPs. Nevertheless, only 47 CNVs (21.07%) were in strong correlation (*r*^2^ ≥ 0.8) with at least one SNP in all five tested windows. A total of 111 SNPs was in strong correlation (*r*^2^ ≥ 0.8). Consequently, these findings highlight that the Affymetrix 6.0 SNP array is not adapted to identify bi-allelic deletion as the majority of them were not well tagged by the nearby SNPs. Furthermore, we evidenced that the strength of the *r*^2^ value decreases as the distance of the CNVs and the SNP increases (Fig. 6). In order to bring out whether not well-tagged bi-allelic deletions tend to be located in the genomic regions where SNP markers are sparse, we performed a correlation analysis. The Spearman test result suggests the absence of any pattern (Spearman’s rank correlation rho *p* value = 0.56) (Supplementary Fig. 10). Nevertheless, a weak association is present between the correlation (*r*^2^) and the distance of SNP from the CNVs (Spearman’s rank correlation rho = 0.1; *p* value = 1.16 × 10^−3^). However, smaller-sized CNVs were generally in strong correlation (Spearman’s rank correlation rho = −0.21, *p* value = 1.08 × 10^−2^) with more SNPs (Spearman’s rank correlation rho = −0.3, *p* value = 2.09 × 10^−6^) (Supplementary Fig. 11).

The 111 tag-SNPs have been analyzed with RegulomeDB to assess their potential functional effect. Only eight variants (7.14%) are considered known expression quantitative trait loci (eQTL) for genes and thus have been shown to be associated with expression as their RegulomeDB score has been estimated to 1f, affecting significantly the expression of many genes in different tissues (data not shown). Three other tag-SNPs are putative functional variants but without eQTL data, and thus no known direct effect on binding was reported. Their scores were low (2a, 2b, and 3a). Among these 11 functional tag-SNPs, only one (rs7542235) has been described to be associated with advanced age-related macular degeneration, age-related macular degeneration with geographic atrophy, and age-related macular degeneration with neovascularization. Due to the unavailability of clinical information on the participants, these phenotypes could not be confirmed.

Furthermore, only 22 genic CNVs (22/223 = 9.9%) were tagged by array SNPs (*r*^2^ > 0.8). Four bi-allelic deletions are overlapping with OMIM genes known to predispose to the hemolytic uremic syndrome, albinism, hereditary neuropathy, mental retardation, and ventricular fibrillation (Table 5). The CNP147 is in high LD with the rs7542235 SNP. This 71-kb deletion spans the entire *CFHR1* and *CFHR3* locus that is associated with an increased risk of the hemolytic uremic syndrome and a decreased risk of age-related macular degeneration. The one-copy deletion frequency is 44% and the homozygous deletion frequency is 14%.

**Table 5:** Tag-SNPs of bi-allelic deletions.

SV ID	Chr:start–end	Size (kb)	Postion	Gene	Tag-SNP	*r*^2^	Disease/trait (OMIM phenotype number)
CNP147	1:196,731035–196,802,072	71.037	Complete gene deletion	*CFHR1*	rs7542235	0.98	Susceptibility to atypical hemolytic uremic syndrome (235400); reduced risk of age-related macular degeneration (603075)
CNP147	1:196,731,035–196,802,072	71.037	Complete gene deletion	*CFHR3*	rs7542235	0.98	Susceptibility to atypical hemolytic uremic syndrome (235400); reduced risk of age-related macular degeneration (603075)
CNP1627	10:78,257,061–78,260,585	3.524	Intronic	*LRMDA*	rs1873468	0.88	Albinism, oculocutaneous, type VII (615179)
CNP1627	10:78,257,061–78,260,585	3.524	Intronic	*LRMDA*	rs2579759	0.89	Albinism, oculocutaneous, type VII (615179)
CNP1627	10:78,257,061–78,260,585	3.524	Intronic	*LRMDA*	rs1080874	0.91	Albinism, oculocutaneous, type VII (615179)
CNP1796	12:869,109–874,029	4.920	Intronic	*WNK1*	rs12369414	0.82	Neuropathy, hereditary sensory and autonomic, type II (201300); pseudohypoaldosteronism, type IIC, (614492)
CNP1196	7:154,393,171–154,400,833	7.662	Intronic	*DPP6*	rs1387191	0.87	Mental retardation 33 (616311)/paroxysmal familial ventricular fibrillation 2 (612956)
CNP1196	7:154,393,171–154,400,833	7.662	Intronic	*DPP6*	rs6975262	0.9	Mental retardation 33 (616311)/paroxysmal familial ventricular fibrillation 2 (612956)
CNP1196	7:154,393,171–154,400,833	7.662	Intronic	*DPP6*	rs6954883	0.9	Mental retardation 33 (616311)/paroxysmal familial ventricular fibrillation 2 (612956)
CNP1196	7:154,393,171–154,400,833	7.662	Intronic	*DPP6*	rs6975879	0.9	Mental retardation 33 (616311)/paroxysmal familial ventricular fibrillation 2 (612956)

We also investigated the potential role of bi-allelic deletions in their etiology of complex diseases or traits. Therefore, we calculated the correlation *r*^2^ between bi-allelic deletions and the SNPs available on the NHGRI-GWAS catalog. Only one GWAS-SNP (rs4704970) was found to be in high correlation with one CNP (CNP877) among the SNPs that have been found to be associated with various complex diseases and traits in the NHGRI-GWAS catalog. Therefore, we redefine our threshold to *r*^2^ > 0.5. Only three SNPs in high correlation (*r*^2^ > 0.68) with three CNVs were identified to be correlated with three diseases or phenotypes, such as high-density lipoprotein cholesterol and triglyceride levels, multiple sclerosis, and obesity (Table 6). One SNP was in perfect correlation (*r*^2^ = 1) with one CNP. The rs4704970 was in perfect correlation with the CNP877. This CNV locus is a 5.957-kb deletion located in chromosome 5 and is located 18.263 kb from the SNP. No RefSeq genes overlap with this locus. The frequency of this deletion in the Tunisian population was high (24.75%), of which 23.76% were one-copy deletions and 0.99% were two-copy deletions. The remaining CNVs, CNP158 and CNVR_16_6_CNP2150, were also found in high LD with two SNPs (rs4844913 and rs11639988, respectively) known for their association with metabolic phenotypes.

**Table 6:** Correlation between bi-allelic deletions and GWAS-SNPs at *r*^2^ > 0.5.

SV ID	Chr:start–end	Size (kb)	GWAS-SNP	*r*^2^ value	GWAS-SNP location	Complex disease/trait
CNP158	1:2,100,816–2,100,839	2.371	rs4844913	0.79	Intergenic	HDL cholesterol and triglyceride levels
CNP877	5:15,5476,772–155,482,729	5.957	rs4704970	1	*SGCD*	Multiple sclerosis (age of onset)
CNVR_16_6_CNP2150	16:19,945,650–19,967,362	21.712	rs11639988	0.68	*GPRC5B*	Obesity

## Discussion

We present a pioneering study of the CNVs in the Tunisian population and provided for the first time a comprehensive map of these structural variations. In agreement with previous studies available from various cohorts and populations, our findings suggest that a high proportion of individuals (75.5%) carried at least one CNV^21,27,37^. We consistently identified reliable 1083 loci spanning 61.443 Mb of the Tunisian genome. It was shown to have more deletions than gains, while deletions seem to be shorter than duplications. Similar results have also been detected in other reports using different arrays, methods, and algorithms^8,23,27,38^. Such patterns have been associated partially with the bias of SNP genotyping arrays for detecting a greater number of deletions than duplications^39,40^. In addition, many detected CNVRs were featured by both losses and gains. These “mixed” loci potentially reflect recurrent copy number changes at the same locus.

The two used programs, PennCNV and Birdseye, implementing the hidden Markov model (HMM) algorithm, show better detection performance among the tools tested on the Affymetrix platform^41^. Nevertheless, we observed large variations in the generated calls, on which we applied a number of quality filtering criteria to minimize false positives and identify high-quality calls. It has been recommended that using multiple CNV calling algorithms and softwares designed for a genotyping platform instead of just one is a better strategy to decrease the false-negative rate as well as to consider subsequently overlapping regions for downstream analysis^37,42^. In addition, for multi-allelic CNVs like those encompassing the salivary amylase and UDP-glucuronosyltransferase genes, obtaining accurate genotypes is particularly challenging. This is observed not only for genotyping array technologies but also using sequencing techniques^43^. The availability of high-throughput sequencing (or next-generation sequencing (NGS)) projects would produce improved CNV calling standards, like the 1000 Human Genomes project and other new efforts^16,44–46^. Large inconsistencies in the outputs of diverse CNV calling algorithms draw special attention to the importance to standardize array data collection, assessment of quality, and experimental validation. Consequently, in order to shed light on the burden of the CNVs on both disease susceptibility and normal phenotype variability, cautious experimental designs as well as accurate data filtering would be required. Hybrid approaches combining NGS and complementary analysis tools will help undoubtedly and correctly define the CNV structure and clear up its function.

In the absence of a Tunisian reference genome, our study gave important insights on CNV distribution that could be of a great impact on public health in Tunisia. In our sample, seven of the reported CNVs, which were all singleton CNVs, were not present in former data submitted to databases such as DGV, 1000 Genomes, gnomAD, and dbVar databases. Taking into consideration the relatively modest size of the studied sample, it is difficult to confirm the specificity of the identified events to the Tunisian population and to determine whether these singleton CNVs are true “rare event” or not.

Furthermore, the comparative analysis of our data with 1000 Genomes data gives insights on the Tunisian population structure. The pairwise correlation analysis suggests that the CNV profile of the Tunisian population is similar to the European and different from the African. Population stratification using principal component analysis with CNP genotype data shows that the Tunisian population clusters with European populations and differs from the African (Romdhane et al., unpublished). These results are in agreement with previous studies on the Tunisian population structure using different autosomal, Y chromosomal, and mitochondrial markers highlighting the heterogeneity of the Tunisian population and the contribution of the European in its genetic background^47–51^.

In general, CNVs can be categorized as pathogenic or benign. These latter CNVs are not often associated with detectable phenotypic outcomes as they overlap with non-functional genomic segments. Functional annotation of the reported CNVs has unveiled notable features as approximately half of the CNV in the Tunisian genome (48.38 %) affected known genes, suggesting that they could contribute to key traits in Tunisian individuals and might affect population health. Mapping genic CNVs to diseases highlighted the enrichment of three major diseases groups, which suggests that Tunisians are likely at risk of developing diseases of the nervous system, congenital malformations, chromosomal abnormalities, and endocrine, nutritional, and metabolic diseases if we take into account our previous results on genetic disease spectrum in the Tunisian population^33^.

The genic CNV segments were found to encompass coding and functional elements, several disease-related genes, and important drug-metabolizing genes that might contribute to the burden of chronic diseases. Homozygous deletions in our dataset are significantly gene poor as it has been previously reported^8^. It has also been shown that rare CNVs harbor more genes than common events^8^. Such relationship was not found in our study mainly due to the modest sample size that underpowered discovering such a pattern. We have also noticed an increased burden of CNVs in some Kyoto Encyclopedia of Genes and Genomes (KEGG) pathways involved in infectious disease susceptibility. Among these genes, the *KIR3DL1* gene that encodes killer cell immunoglobin-like receptors (KIR) was identified in a “mixed” locus. It has been reported that the counts of individual genes in the KIR locus directly influence early aspects of HIV-1 control^52^. This finding deserves further investigations in order to understand different degrees of susceptibility and resistance to HIV infection in Tunisia and other the Middle East and North African (MENA) populations^53,54^.

Furthermore, we noted enrichment in other medically relevant pathways, including starch and sucrose metabolism, carbohydrate digestion and absorption, and biosynthesis of unsaturated fatty acids. Among genes involved in such pathways were the salivary amylase gene *AMY1* and the pancreatic amylase gene *AMY2* for which copy number changes were previously shown to be determinant of metabolic states, such as body mass index, obesity, and insulin resistance^55,56^. These metabolic markers are present in diabetes, a serious population health problem that shows the highest prevalence in the MENA region with >10% of the population being diabetic^57^. Therefore, it would be preferable to investigate whether the amylase genes associated with diabetes in our population in addition to the predisposing genetic factors already described in Arabs^58,59^. Moreover, *MGAM* is another gene involved in such metabolic pathways and was associated with non-syndromic oral clefts, a common birth defect^60^. Deletions found at this gene should be further investigated as biomarkers of such congenital malformations in our population.

In addition, CNV genes are also enriched in drug metabolism and detoxification, as well as chemical carcinogenesis pathways. The most common example is *UGT2B17*, where CNV has been correlated with pancreatic, prostate, and colorectal cancer, in addition to other diseases related to differential testosterone concentrations^61–63^. *UGT2B17* gene deletion (UGT2B17*2) has been associated with the pharmacokinetics of aromatase inhibitor drugs such as exemestane, as well as in bone health^64^.

It is important to recall that all genotyped individuals were phenotypically healthy at the moment of recruitment. Therefore, the possibility that the carried CNV might be responsible for a disease cannot be excluded in the absence of complete prospective phenotypic data as we observed a high proportion of deletions in coding sequences, even complete gene losses at homozygous states. Common polymorphic deletions have been essentially identified in coding exons of genes involved in sex steroid metabolism (*UGT2B28* and *UGT2B17*), olfactory receptors such as *OR51A2* and *OR4F5*, and drug response, namely *CYP2A6*, *GSTT1*, and *GSTM1*^65^. It has been suggested that these common deletion polymorphisms generally depict ancestral mutations being in LD with nearby SNPs^65^. Interestingly, non-coding sequences such as microRNAs and introns were found to overlap with several of our CNV loci. Consequently, they could affect different biological process regulation such as cell apoptosis, proliferation, and differentiation. MicroRNAs have also been linked to human diseases. Indeed, some studies have evidenced downregulation of microRNAs in tumors when compared with normal tissues as well as their upregulation expression due to CNV in lymphoma^66,67^. Moreover, it has been recently evidenced that intronic CNVs influence gene expression variability and splicing^68^. Therefore, the CNV overlapping coding genes, introns, and microRNAs found in our study could not only contribute to genetic diversity but also in disease susceptibility in our population.

The presence of CNVRs spanning functional sequences, including those associated with the disease, challenges the discrimination between benign and pathogenic CNVs^69^. Apparently, healthy individuals harboring deletions of partial and/or complete coding exons in genes known to be responsible for severe genetic diseases remain to be understood, as medically relevant genes harboring homozygous deletions have been identified in our study. Homozygously deleted genes have also been reported in the CNV map generated by Zarrei et al.^69^, involving OMIM genes such as *UGT2B17*, *RHD*, *KIR3DL1*, *PSG1*, *HLA-DRB1*, and *HLA-DQA1*. These genes were considered as non-essential and “dispensable” because they could be absent from the genomes of apparently healthy individuals^15,69^.

The assessment of the burden of “pathogenic” CNVs on health is challenging due to variable phenotypic manifestations between individuals harboring similar CNVs. A “double-hit” model has been advanced in order to explain the variable expressivity involving the 16p11.2 microdeletions and duplications^70–74^. These CNVs have been previously described in neurologic diseases like mental retardation, schizophrenia, and autism, and even in apparently healthy individuals^70–74^. In their study, the authors advanced that a first hit, such as the 16p12.1 microdeletion, in combination with a secondary hit like an epigenetic, environmental, or genetic insult could result in a more severe phenotype^75^. Phenotypic effects of CNVs on individuals could be influenced by several properties.

It has been reported that inherited CNVs are more likely to be benign, while those pathogenic tend to be enriched for de novo mutation^76,77^. In addition, the position of the CNV within the genome has a significant influence on the phenotype. Indeed, CNVs spanning genes important to development, dose-sensitive genes or regulatory sequences, are likely contributing to disease expression or predisposition. The production of a functional transcript has been evidenced after a gain or a loss of genetic material and depends on different gene features such as the coding phase of the non-affected exons and the presence of alternative splicing isoforms that may counterbalance for the depletion of the major transcript, as it has been illustrated by the study on the *NRXN1* gene involved in schizophrenia^78^.

Moreover, pathogenicity also appears to be proportional to the size of the CNV, as large CNVs likely affect multiple genes, whereas smaller CNVs affect fewer genes. Furthermore, the nature of the CNV itself is also mandatory for pathogenesis as duplications are noticed to have a smaller pathogenic burden than deletions^79^. This is the case for the SMN1 locus known to cause AR disease spinal muscular dystrophy. Deletions of this locus are found in 96% of SMA patients^80^, whereas persons harboring two or more copies of the *SMN1* gene are typically healthy^81,82^. Decoding the complex relationship between CNV genotypes and apparent phenotype is challenged by confounding elements such as environmental factors and variable penetrance. CNV-associated phenotypes can be impacted by haploinsufficiency, genomic imprinting, and the presence of other genetic factors^77^. All these findings emphasize the importance of decoding this type of variation to decipher the required part of the genome for normal human development due to the gene function loss^83–86^. Our study is the first step in building a Tunisian-specific structural variation database, thus paving the way to assess the burden of rare and common CNV of the Tunisian “CNVariome” in a much larger cohort^87^.

In an additional analysis, we examined SNPs that are in high LD with CNVs. We took only 223 bi-allelic deletions. These variants (tag-SNPs for CNV) would serve to predict CNV alleles when genotyped and proxy CNVs in investigating associations between CNVs and disease. Such an approach would reduce genotyping costs because SNPs are presently much easier to genotype than CNVs. Some reports supposed that deletion polymorphisms are commonly in strong LD and segregate on ancestral SNP haplotypes^65,88^. Tagging SNPs were found for only 146 CNVs (65.76%). By this analysis, we aimed also to determine the usefulness of the Affymetrix 6.0 SNP chip to identify CNVs in the Tunisian population. The efficacy of this array was rather poor, with 40.54% of CNVs that were tagged at *r*^2^ > 0.5 and 21.17% in strong LD. Similar findings were observed in the CNV study in the Qatari population when using the Illumina OMNI2.5M array^16^. This taggability gap could be attributed to the local SNP density paucity, thus influencing the LD level. A comparative LD analysis using two different SNP sets genotyped by different SNP arrays revealed that ~80% of CNVs are in high LD (*r*^2^ > 0.8) when HapMap phase 2 SNP set was used compared to ~50% of CNVs that were in high LD with commercial SNP array sets^18^. An improvement of this taggability has been obtained with whole-genome sequencing SNPs as >70% of the deletions have been tagged by at least one SNP at *r*^2^ > 0.5 and over 50% at *r*^2^ > 0.8 in the Qatari dataset in agreement with LD data generated using 1000 Genome dataset^15,16^. Consequently, this fact suggests that CNV genotyping could be challenging using this or other commercial arrays in North African populations. Furthermore, correlation analysis between bi-allelic deletions and GWAS-SNP indicates that these loss segments could be likely causal variants because of their strong LD with GWAS-SNP. Notably, the high correlation between the CNP877 and the GWAS-SNPs near the *SGCD* gene involved in multiple sclerosis is consistent with a previous study^27^. Consequently, data on LD between SNPs and deletions highlight the advantage of a unique database integrating findings on SNP genotypes and structural variations and could be combined in eventual arrays conceived to genotype Tunisian cohorts, in addition, to imputing these deletions in Tunisian or in ancestrally and/or ethnically similar populations.

In summary, we provided the first genome-wide study of CNVs leading to a CNV map of the Tunisian population by generating a highly dense catalog of 1083 CNVRs in a cohort of 102 Tunisians using stringent QC criteria for CNV detection. Our study contributes to shedding light on this unstudied kind of variation in the Tunisian population. CNV genes reported here are involved in biological pathways relevant to public health. In addition, we brought out the first assessment of LD between bi-allelic deletions and SNPs in the Tunisian population, thus allowing their imputation in future studies of a matched cohort. Knowing the prevalence and characteristics of recurrent CNVs is clinically invaluable. Consequently, they should deserve further characterization and be systematically assessed in a larger cohort in order to assess significance and associations with the relevant diseases or traits in the Tunisian population. This specificity will be informative for the design of a population-specific clinical copy number array, the interpretation, and the assignment of the pathogenicity of such variations within our population. These findings gave first insights into the CNVariome of the Tunisian population and raise many questions regarding their contribution to health issues in the Tunisian population and ethnically similar North African populations, thus paving the way to precision medicine implementation in the MENA region.

## Methods

### Study population sample preparation and genotyping

A total of 135 healthy (free from any genomic disorder) unrelated Tunisian individuals (103 males and 32 females) originating from Northern, Central, and Southern Tunisia has been recruited. Participants’ mean age is 48 ± 10 years. All individuals gave informed consent. Identities of the participants were kept anonymous and no personal identifiers were used. According to the Declaration of Helsinki Principles, ethical approval was obtained from the biomedical ethics committee of Pasteur Institute of Tunis (PV09/06, IRB# 0,000,000,044).

All samples have been genome-wide scanned using the Affymetrix Genome-Wide SNP Array 6.0 as mentioned in a previous study^89^. This array contains 906,600 polymorphic probes designed to detect both SNPs and CNVs as well as 946,000 non-polymorphic probes to call CVNs only. CNV probes were basically chosen for their genomic spacing and based on known CNVs available in the DGV (Affymetrix Inc.: Genome-wide human SNP array 6.0 Datasheet. Available at www.affymetrix.com 2009). Sixteen individuals were excluded from subsequent analysis as they had QC contrast values >0.4. The remaining 119 individuals were used for CNV detection.

Affymetrix Power Tool (APT) v1.8.6 was used to obtain genotype calls required for copy number estimation. Called SNPs were excluded if they do not fit the following criteria: minor allele frequency (MAF) >1%, genotype rate >95%, and *p* value for Hardy–Weinberg equilibrium (HWE) test over 10^−4^. This quality control step was performed using PLINK v1.07^90^ leaving a total of 782,392 SNPs for subsequent analysis.

### CNV detection algorithms and analyses

The two most used CNV detection algorithms, namely PennCNV v.1.0.3^91^ and Birdsuite v1.5.5^92^, were used for both CNV detection and validation. In this study, we only focused on CNVs in the 22 autosomes because of the inaccuracy of CNV detection in sex chromosomes. Genomic coordinates for all CNVs detected in this study were mapped to the human genome assembly build 37 (hg19).

In the first step, CNV segments were identified using the HMM algorithm implemented in PennCNV. We followed the PennCNV-Affy Protocol available at this link (http://www.openbioinformatics.org/penncnv/penncnv_tutorial_affy_gw6.html). The Log 2R and B-allele frequency values were obtained. PennCNV provided CNV-specific quality control (QC) metrics in order to find potentially poor CNV samples. Individuals with poor quality of signal intensity were removed if they had >100 CNV segments detected, along with wave factor >0.05 or Log 2R standard deviation >0.4 and B-allele drift >0.0125. The wave factor consists of the overall “waviness” or variation of signal intensity and the B-allele drift is the fraction of “abnormal” markers not clustering in the usual positions (0, 0.5, and 1). This value represents the median of all chromosomes and it is useful for detecting genotyping failure^42^.

In order to assess the copy number changes, we used the HMM model. It mainly executes segmentation of the log 2 ratio intensity data. In addition, it predicts copy number states for each segment. There are up to five delineating the following states: CN state = 0 (homozygous deletion), CN state = 1 (heterozygous deletion or single-copy loss), CN state = 2 (neutral copy number of normal diploid), CN state = 3 (single-copy gain), and CN state = 4 (amplification). CNVs with a normal copy (state = 2) are not incorporated in the final CNV report. In addition, we used a minimum number of ten SNPs overlapping with the CNV in order to prevent false-positive CNV. We used also the same algorithm to merge adjacent CNVs because it splits large CNV (>500 kb) into smaller parts between 100 and 150 kb CNV calls. Spurious CNV calls in specific genomic regions (immunoglobulin, telomeric, and centromeric) were removed.

Along with PennCNV, we used the Birdsuite package^92^ to detect copy number change. There are two components in Birdsuite. The first one is Birdseye, which is able to call rare copy number changes. As such, the CNVs detected by the two algorithms could be cross-validated on the 102 samples. Birdseye called 96,177 CNVs. Those with low confidence scores (<5), as recommended by the program, were excluded from subsequent analysis. Similarly, only CNVs on autosomes were used and we restricted the number of SNPs overlapping with each CNV to three markers. Therefore, 6670 CNVs with a normal copy are not included in the final CNV report. CNVs called in centromeric regions are likely to be false positives because SNP coverage in these regions is very low. Consequently, all the CNVs spanning centromeres, telomeres, and immunoglobulin regions were excluded from the analysis for both algorithm outputs. In addition, we excluded CNVs <1 kb or >3 Mb, leading to a final set of 6263 CNVs.

The genotype of 1316 CNPs can be defined by the second component of Birdsuite, which is a Canary used to determine the integer copy number state at each of these CNPs predefined on the Affymetrix 6.0 array^92^. These CNPs are distributed on all the autosomes as well as the heterochromosomes. They were identified in more than one HapMap II individuals and their sizes were also precisely determined^92^. The Canary algorithm was performed on the 102 Tunisians. As recommended by the software, only CNP on the 22 autosomes were kept for subsequent analysis as well as those with integer copy numbers detected with a high confidence score (>0.1). CNPs were filtered according to their size, only those ≥1 kb were kept.

### Construction of CNV loci using PennCNV and Birdseye outputs

The CNVs identified by both PennCNV and Birdseye overlapped across individuals. Therefore, they were merged into discrete non-overlapping loci called CNVR. The boundaries of each locus were assessed by the union of all CNVs that are included in that given locus. A CNVR is then defined as the maximum region that is shared between all individuals harboring a CNV at the same locus. This step was achieved using the BedTools v2.25.0 utilities^93^. The CNVRs were classified into three classes, “loss” (loci encompassing deletions), “gain” (loci encompassing duplications), and “mixed” (loci encompassing both deletions and duplications). The CNV locus construction was performed in order to assess the CNV frequencies in the studied population.

### Comparison of CNVRs detected by PennCNV and Birdseye

The CNVRs generated using the PennCNV and Birdseye outputs were compared as a concordance and in silico “validation” step. The reciprocal 50% overlapping method was used to compare the CNVRs identified by these two computational methods. CNVRs found to overlap with 50% of their lengths were considered as CNV locus and kept for further analyses. This final set of CNV was called vCNVRs.

### Construction of the global CNV map and comparison to structural variants from the 1000 Genomes Project and gnomAD

In order to construct the comprehensive autosomal CNV map in the Tunisian population, we merged vCNVRs and Canary results to generate the global CNV map. To further provide reliable data, we downloaded the 1000 Genomes phase 3 structural variant data in VCF format as reported in the original publication^15^ (https://www.internationalgenome.org/) as well as the structural variant dataset from the gnomAD^94^. We then compared our CNVR dataset while requiring at least 50% of reciprocal overlap size using Bedtools. When CNVR candidates in our dataset matched multiple allele structural variants in these databases, we summed the frequency of all alternate alleles. CNVR with frequency >90% not overlapping with any structural variants from these databases were considered as potential false positive and removed. Pairwise correlation analysis of frequency between the CNV frequency was identified in the present study and that of the 1000 Genomes. The *p* value threshold for statistical significance is 5%.

### Functional annotation of the global CNV map

Merged vCNVRs and allelic CNP of the global CNV map were searched to find overlapping genes using the RefSeq Gene annotations (the hg19 genome version) using the reciprocal 50% overlapping threshold. These genes were screened against the OMIM database to find out whether these copy number segments mapped to disease and phenotype genes. In addition, we also obtained disease–gene associations available from the GAD through the DAVID 6.8 bioinformatics suites in order to predict disease–metabolic pathway associations^84,95^. The GAD is the NIH-supported public collection of human genetic association studies of complex diseases^36^. This database includes the complete known gene–phenotype associations as well as non-Mendelian common complex diseases^36^. Furthermore, we considered gene ontology of the overlapping genes with this CNV map in order to estimate the enrichment of these genes when compared with other genes of the human genome using the DAVID bioinformatics resources^95^. In addition, pathway analysis of these genes was considered using the KEGG pathway database by the DAVID bioinformatics suite. The statistical significance cutoff was 0.05. In addition, the WHO ICD-10 version 2007 was used (http://apps.who.int/classifications/apps/icd/icd10online/) in order to determine the genetic disorders distribution according to the affected tissue, process, system, or organ.

### Identification of novel CNV loci

In order to establish whether a CNV locus is novel, we have compared our data to those available in the following databases: DGV, dbVar, the DDD study^96^, the study of Ira M. Hall’s lab (IMH)^97^ using the AnnotSV v2.3 software^98^ in addition to those in 1000 Genomes, and gnomAD. A CNV was designed as a novel if it did not share at least 50% of its size with any CNV loci stored in these databases.

### LD analysis

We performed a correlation analysis for CNV and nearby SNPs within five windows (200 kb, 500 kb, 1 Mb, 2 Mb, and 3 Mb). Called SNPs were previously subject to exclusion if they do not fit the following criteria: MAF over 1%, genotype rate over 95%, and *p* value for HWE test over 10^−4^. LD analysis was performed using the “--r2 --ld-snp” PLINK v1.07 command^90^. In this LD analysis, a bi-allelic model was applied as we only selected polymorphic bi-allelic deletions (frequency ≥ 4%). We used the squared Pearson’s correlation (*r*^2^) for correlation analysis implemented in the PLINK v1.07 software^90^. If an SNP with a high *r*^2^ (>0.8) for a CNV locus allele was identified, then we designed the CNV allele as being in LD with that single-nucleotide variant. The “tag-SNP” was selected as the variant having the highest *r*^2^ value from such SNPs.

### Functional tag-SNP annotation

We performed a functional annotation of tag-SNPs with predicted regulatory elements using the RegulomeDB database^99^ (http://www.regulomedb.org). DNA regulatory elements encompass regions of DNAase hypersensitivity and binding sites of transcription factors, as well as promoter regions biochemically characterized in transcription regulation. Additional databases like Ensembl (https://www.ensembl.org), ClinVar (https://www.ncbi.nlm.nih.gov/clinvar/), and the NHGRI-GWAS catalog (https://www.ebi.ac.uk/gwas/) have also been queried.

### Statistical analysis and data visualization

All the downstream analyses were performed using the statistical software R version 3.6.2 (http://www.r-project.org). For data visualization, the R package ggplot2 was used^100^. RCirocs was used to plot genome-wide distribution of CNV states^101^.

### Reporting summary

Further information on research design is available in the Nature Research Reporting Summary linked to this article.

## Supplementary information

Supplementary Information

Supplementary Data1

Reporting Summary

## Data Availability

Datasets supporting the conclusions of this article are included within the article, Supplementary Tables, and Supplementary Data. Other data that support the findings of this study are available from the corresponding author on reasonable request. In Tunisia, genetic data are considered as personal private data, for these reasons we have submitted the minimal dataset as supporting files, but we are not allowed to submit the full raw data. The full raw data may be made available upon request by other investigators and after approval of our IRB.

## Code availability

No custom code has been used in this study. Only open source softwares have been used.
