# Supplementary material for: A map of copy number variations in the Tunisian population: a valuable tool for medical genomics in North Africa
Source: NPJ Genom Med. 2021 Jan 8;6:3. doi: 10.1038/s41525-020-00166-5 (PMC7794582; doi:10.1038/s41525-020-00166-5)
Supplement: Supplementary file 1 — Supplementary Information [file 41525_2020_166_MOESM1_ESM.pdf]

TITLE: A map of copy number variations in the Tunisian population: A valuable tool for medical genomics in North Africa

Authors:

Lilia Romdhane[1,2], Mezzi Nessrine[1], Hamza Dallali[1], Olfa Messaoud[1], Shan Jingxuan [3,4,5], Khalid A. Fakhro[6,7], Rym Kefi[1], Lotfi Chouchane[3,4,5], Sonia Abdelhak[1]

Authors' affiliations:

1: Biomedical Genomics and Oncogenetics Laboratory (LR16IPT05), Institut Pasteur de Tunis, Tunis, Tunisia
2: Department of Biology, Faculty of Science of Bizerte, Jarzouna, Tunisia
3: Department of Genetic Medicine, Weill Cornell Medicine, New York, USA.
4: Department of Microbiology and Immunology, Weill Cornell Medicine, New York, USA.
5: Genetic Intelligence Laboratory, Weill Cornell Medicine in Qatar, Education City, Qatar Foundation, Doha, Qatar
6: Department of Genetic Medicine, Weill Cornell Medical College in Qatar, Doha, Qatar.
7: Department of Human Genetics, Sidra Medicine, Doha, Qatar

Corresponding author's contact:

Biomedical Genomics and Oncogenetics Laboratory
Institut Pasteur de Tunis, BP 74, 13 Place Pasteur 1002 Tunis Belvédère Tunisia,
Department of Biology, Faculty of Science of Bizerte, Jarzouna, Université Tunis Carthage, Tunis, Tunisia
Tel: 216 71 849 110
Fax: 216 71 791 833, *E-mail:* lilia.romdhane@fsb.rnu.tn **;**

Supplementary Information Table of Contents:

Supplementary Figures 1-11 ………………………………………………………pp. 3-22

Supplementary Tables 1-10 ………………………………………………………pp. 23-35

Supplementary Figure 1: PennCNV CNV distribution among Tunisian individuals

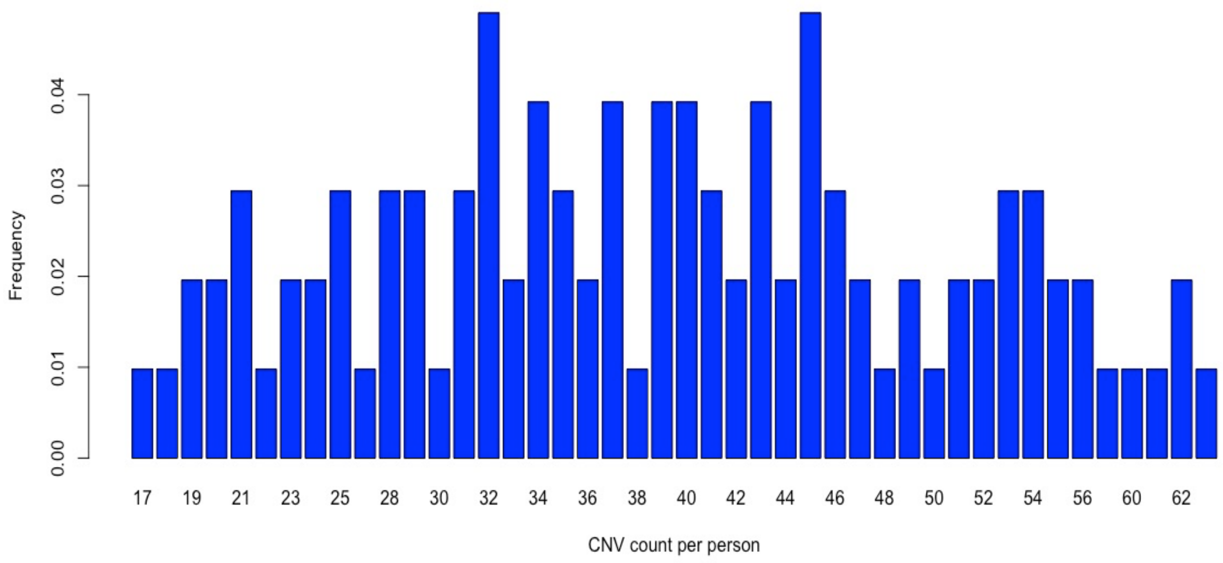

Figure 1: PennCNV CNV count per individual

Supplementary Figure 2a: PennCNV CNV distribution on autosomes

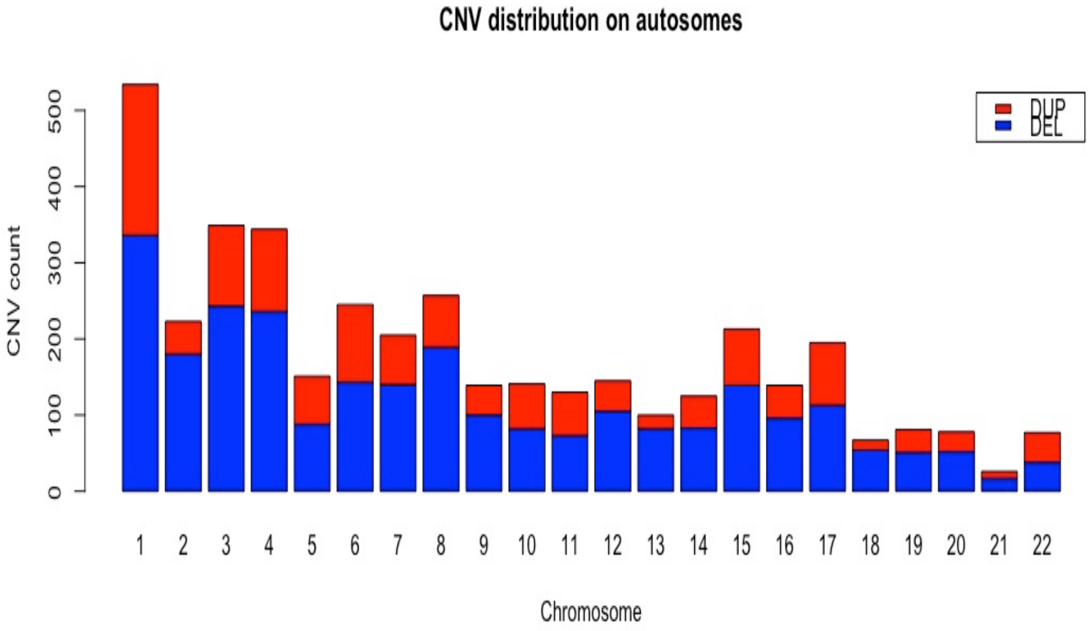

Figure 2a: PennCNV CNV distribution on autosomes

Supplementary Figure 2b: PennCNV CNV size distribution

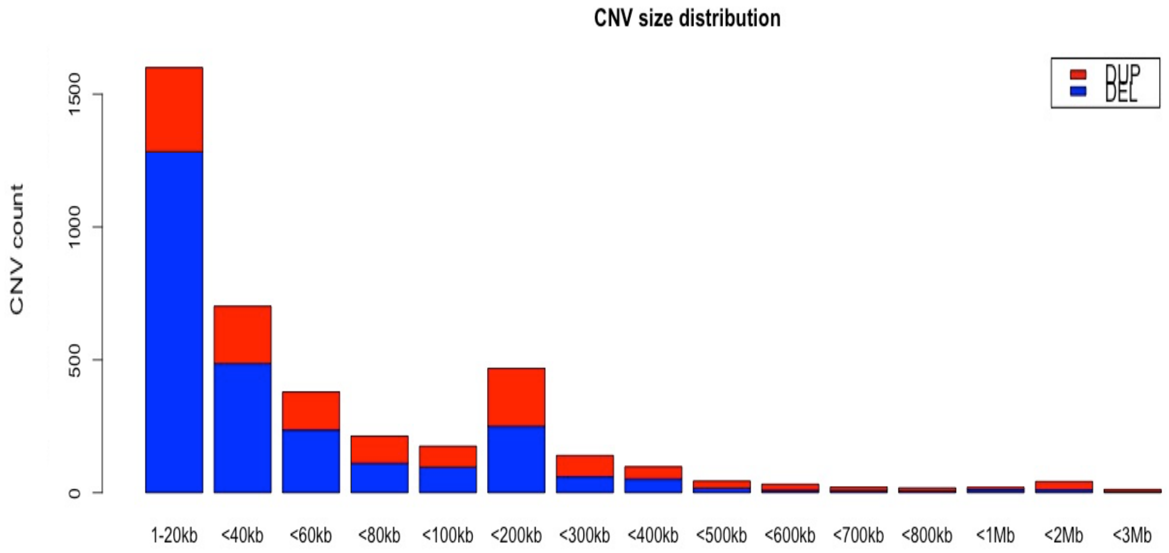

Figure 2b: PennCNV CNV size distribution

Supplementary Figure 2c: Birdseye (Birdsuite) CNV distribution on autosomes

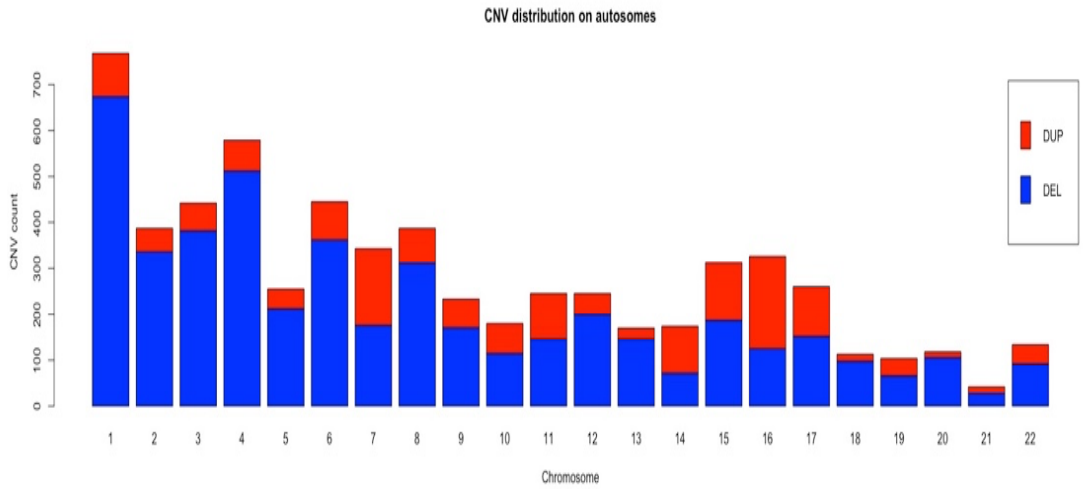

Figure 2c: Birdseye (Birdsuite) CNV distribution on autosomes

Supplementary Figure 2d: Birdseye (Birdsuite) CNV size distribution

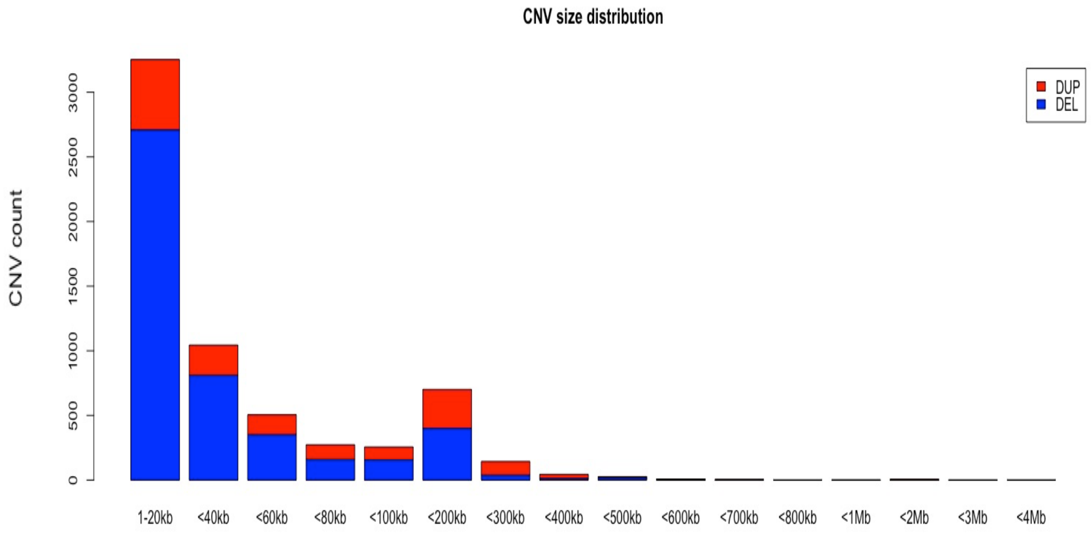

Figure 2d: Birdseye (Birdsuite) CNV size distribution

Supplementary Figure 3a: PennCNV CNVR size distribution

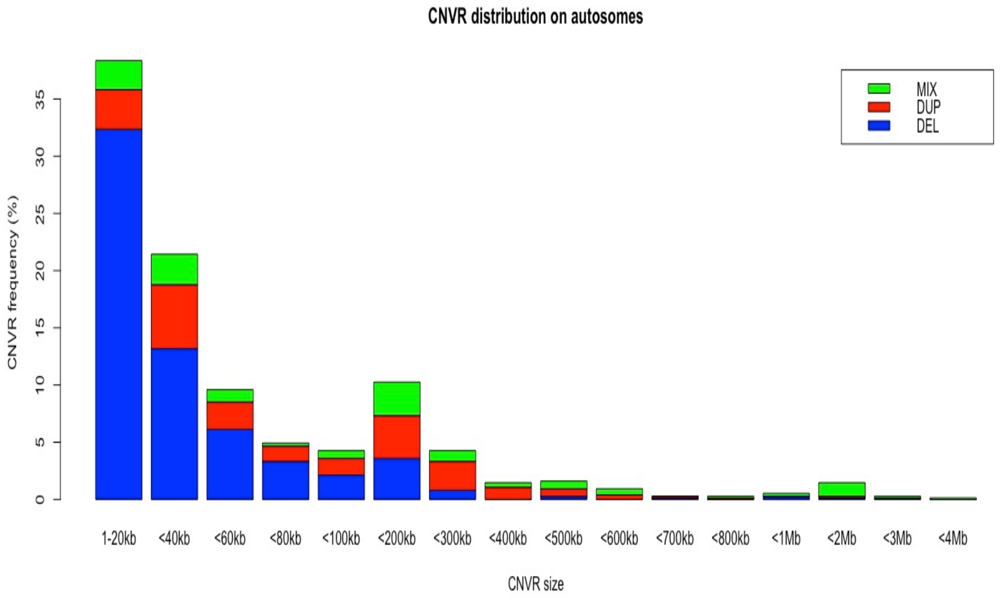

Figure 3a: PennCNV CNVR size distribution

Supplementary Figure 3b: Birdseye (Birdsuite) CNVR size distribution

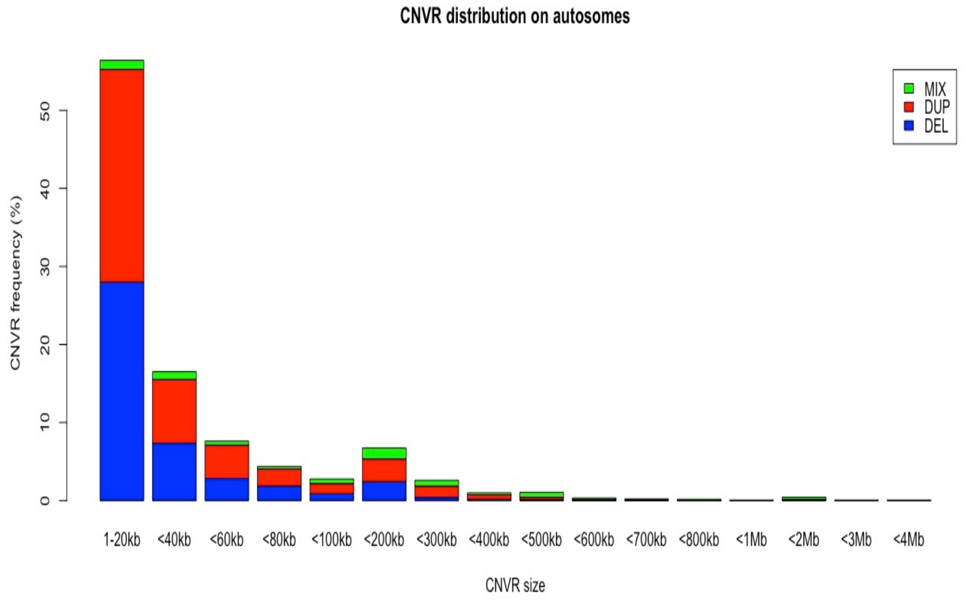

Figure 3b: Birdseye (Birdsuite) CNVR size distribution

Supplementary Figure 4a: Venn diagram showing common vCNVR between PennCNV and Birdseye outputs

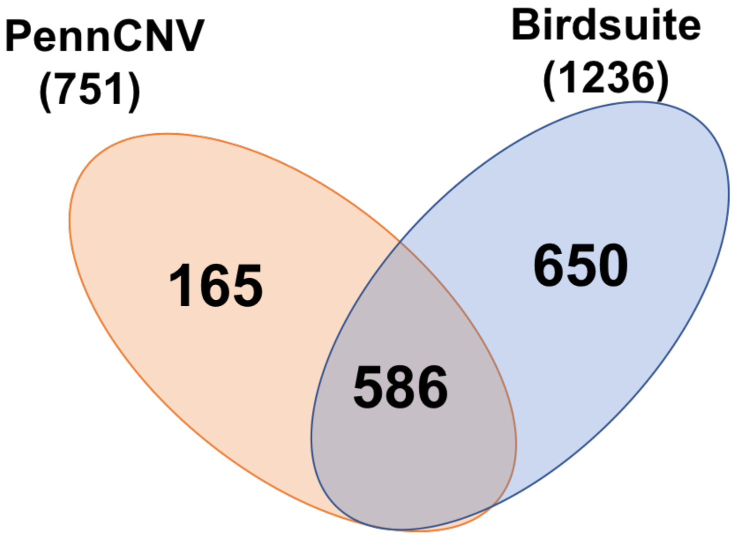

Figure 4a: Venn diagram showing common vCNVR between PennCNV and Birdseye outputs

Supplementary Figure 4b: vCNVR distribution on autosomes

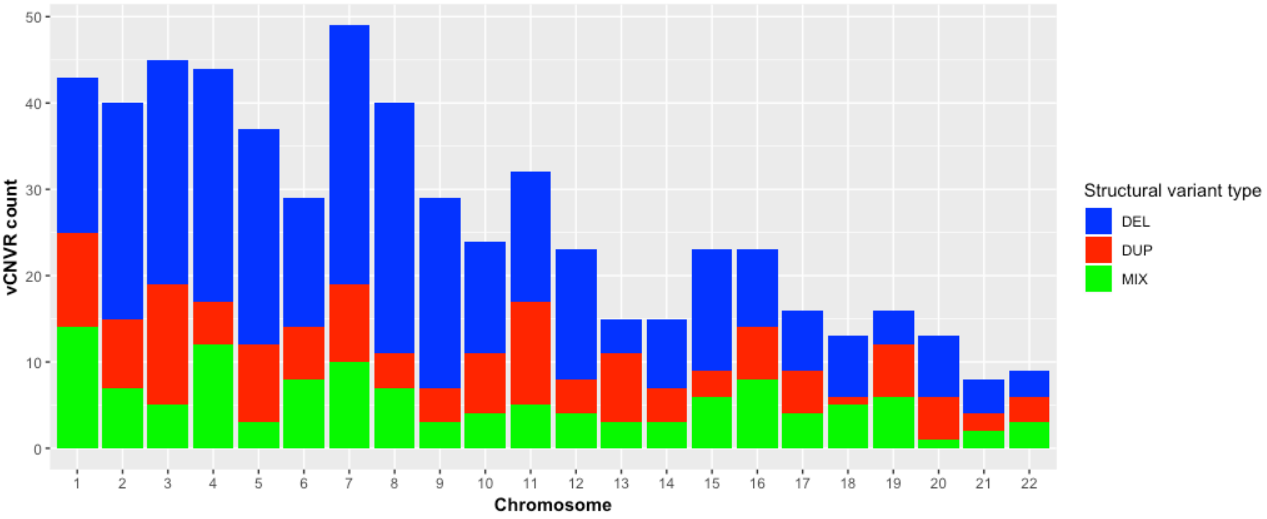

Figure 4b: vCNVR distribution on autosomes

Supplementary Figure 5: vCNVR length distribution

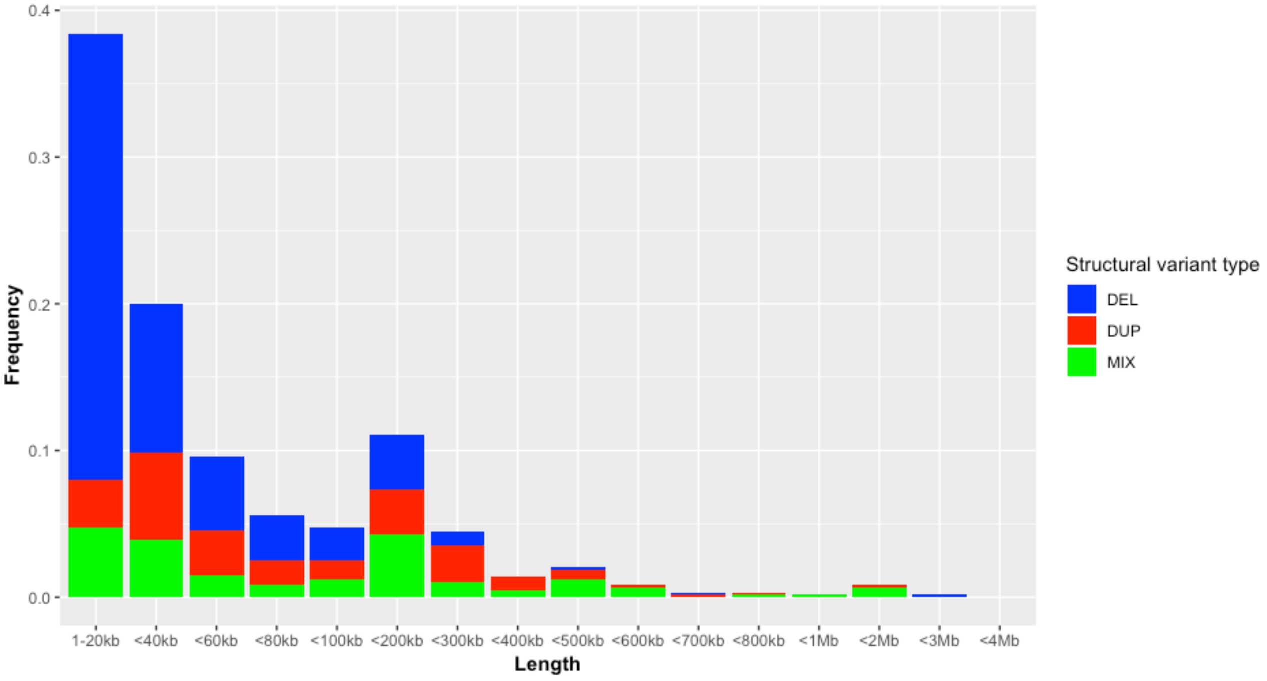

Figure 5: vCNVR length distribution

Supplementary Figure 6: Distribution of CNP on autosomes

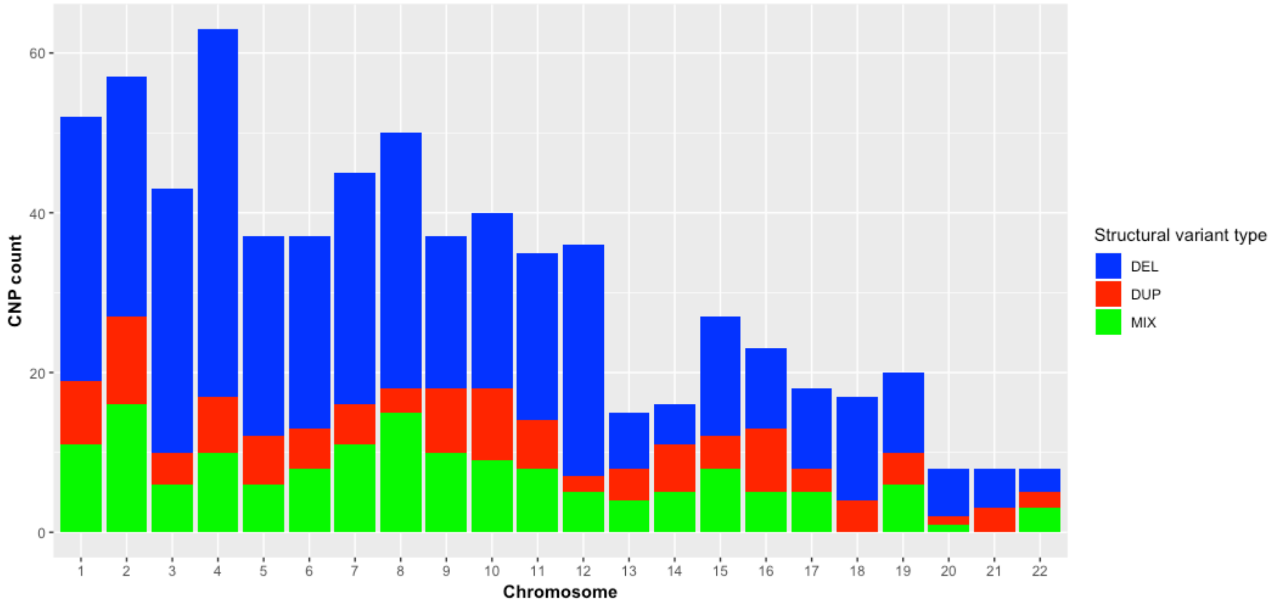

Figure 6: Distribution of CNP on autosomes

Supplementary Figure 7: CNP Size distribution

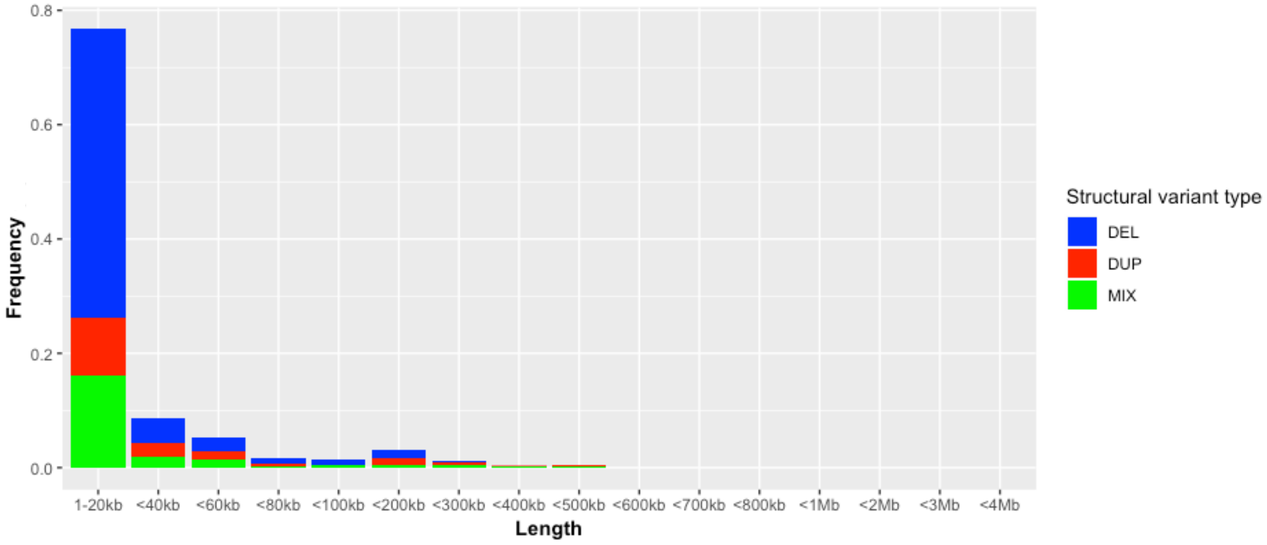

Figure 7: CNP Size distribution

Supplementary Figure 8: Global CNV map chromosome distribution

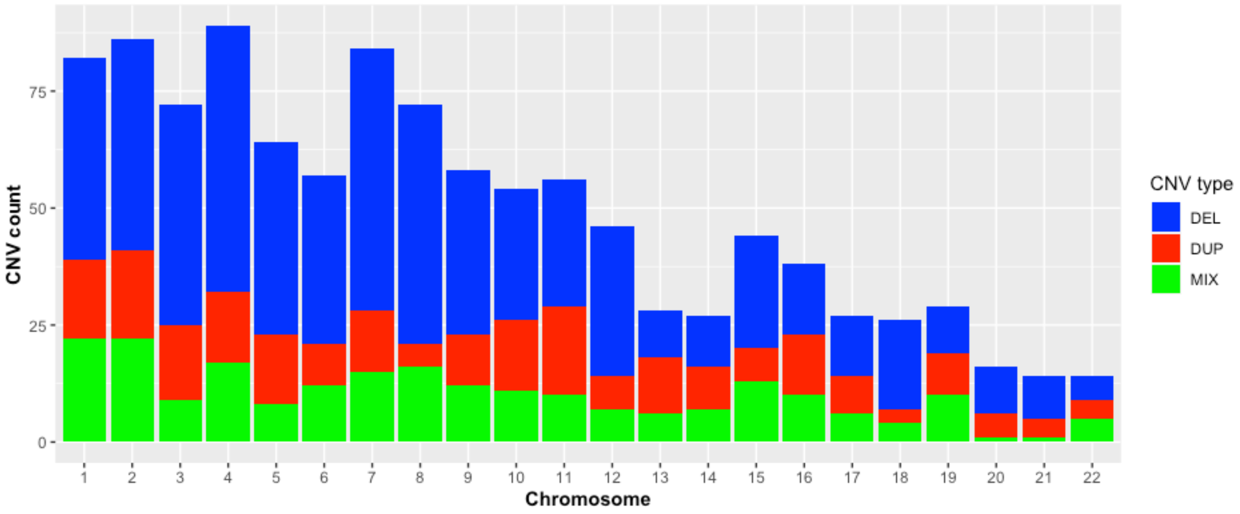

Figure 8: Global CNV map chromosome distribution

Supplementary Figure 9a: Pairwise correlation between CNV frequency in Tunisia and AFR (1000Genomes)

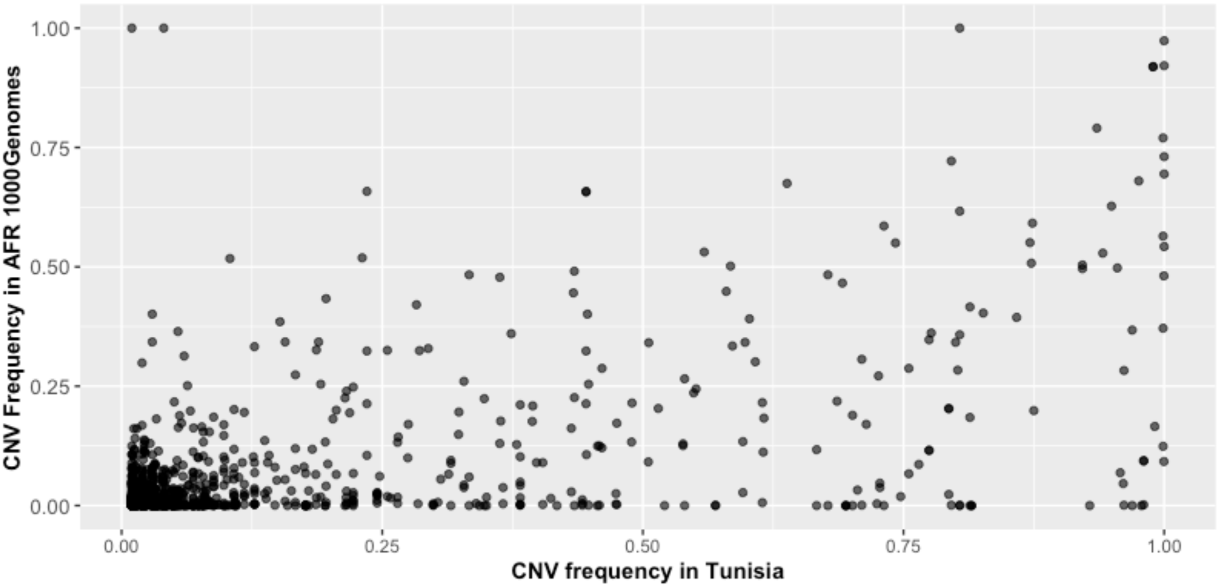

Figure 9a: Pairwise correlation between CNV frequency in Tunisia and AFR (1000Genomes)

Supplementary Figure 9b: Pairwise correlation between CNV frequency in Tunisia and EUR (1000Genomes)

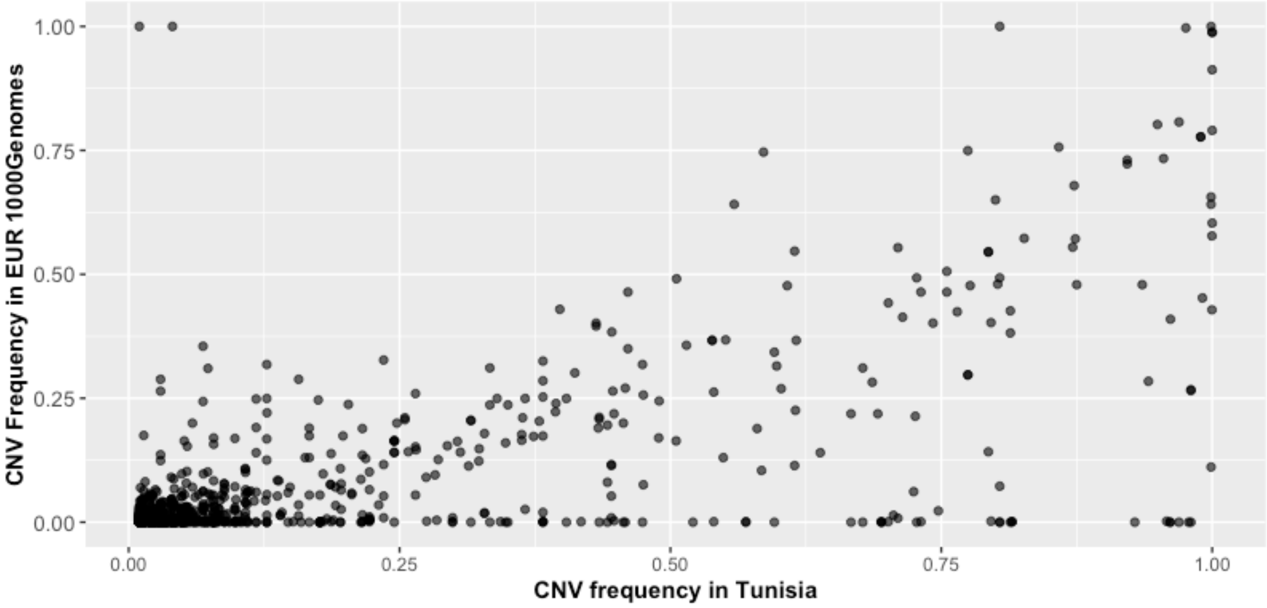

Figure 9b: Pairwise correlation between CNV frequency in Tunisia and EUR (1000Genomes)

Supplementary Figure 9c: Pairwise correlation between CNV frequency in Tunisia and AMR (1000Genomes)

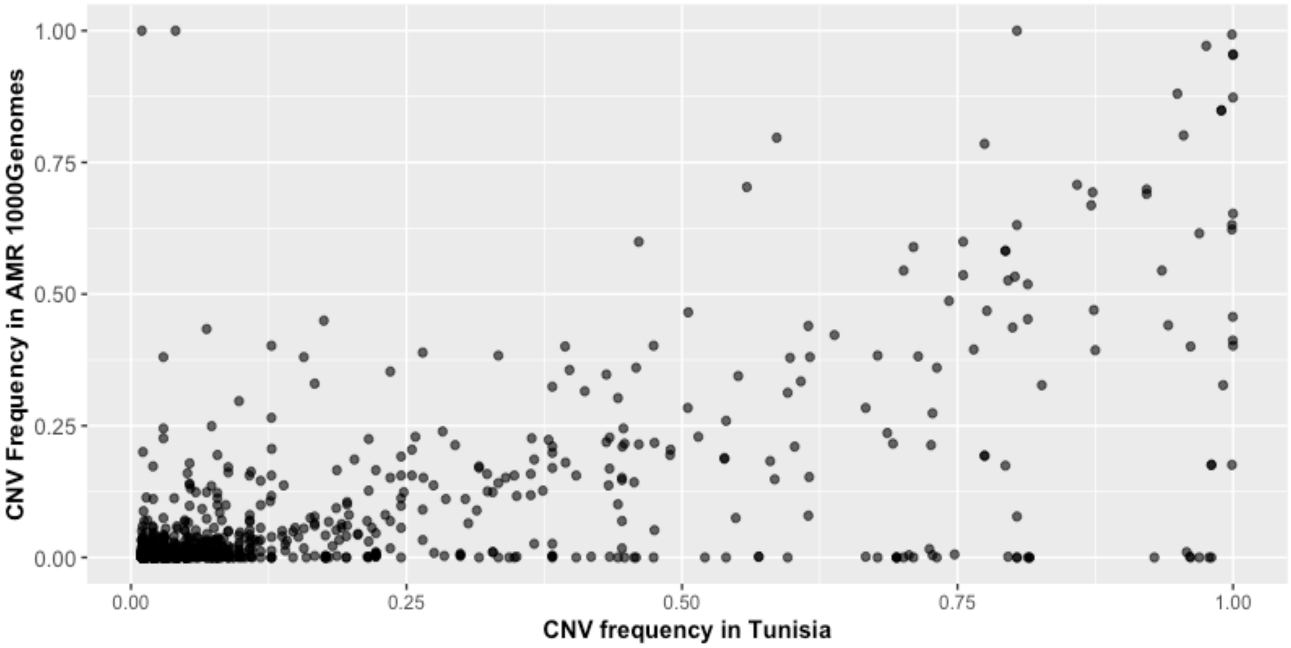

Figure 9c: Pairwise correlation between CNV frequency in Tunisia and AMR (1000Genomes)

Supplementary Figure 9d: Pairwise correlation between CNV frequency in Tunisia and EAS (1000Genomes)

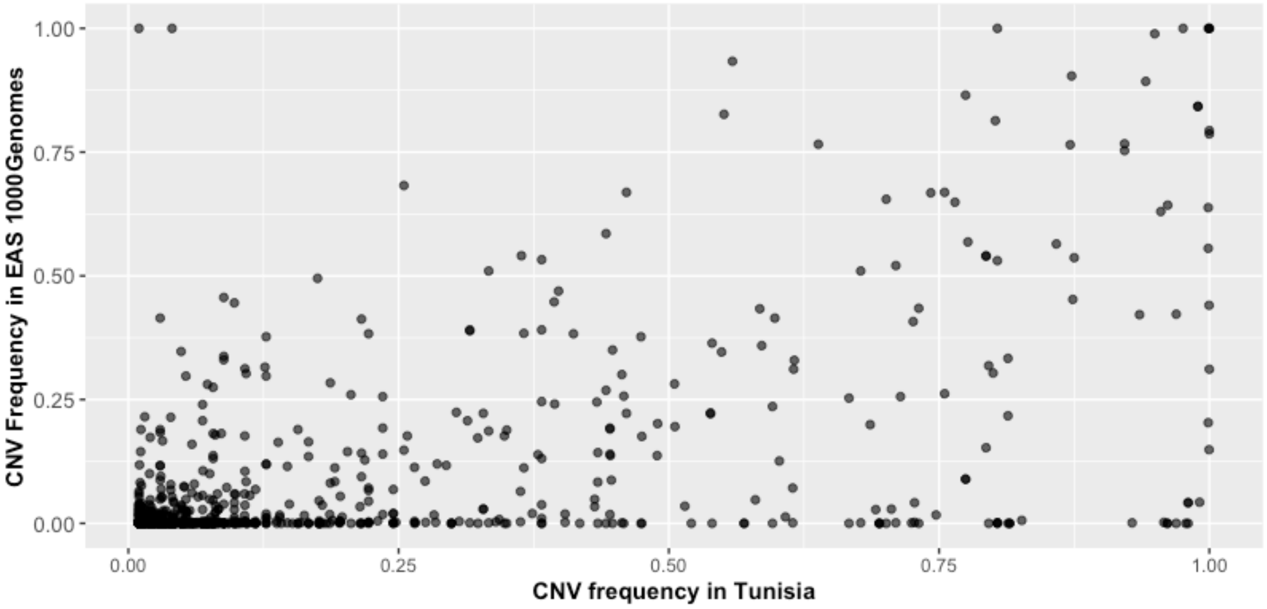

Figure 9d: Pairwise correlation between CNV frequency in Tunisia and EAS (1000Genomes)

Supplementary Figure 9e: Pairwise correlation between CNV frequency in Tunisia and SAS (1000Genomes)

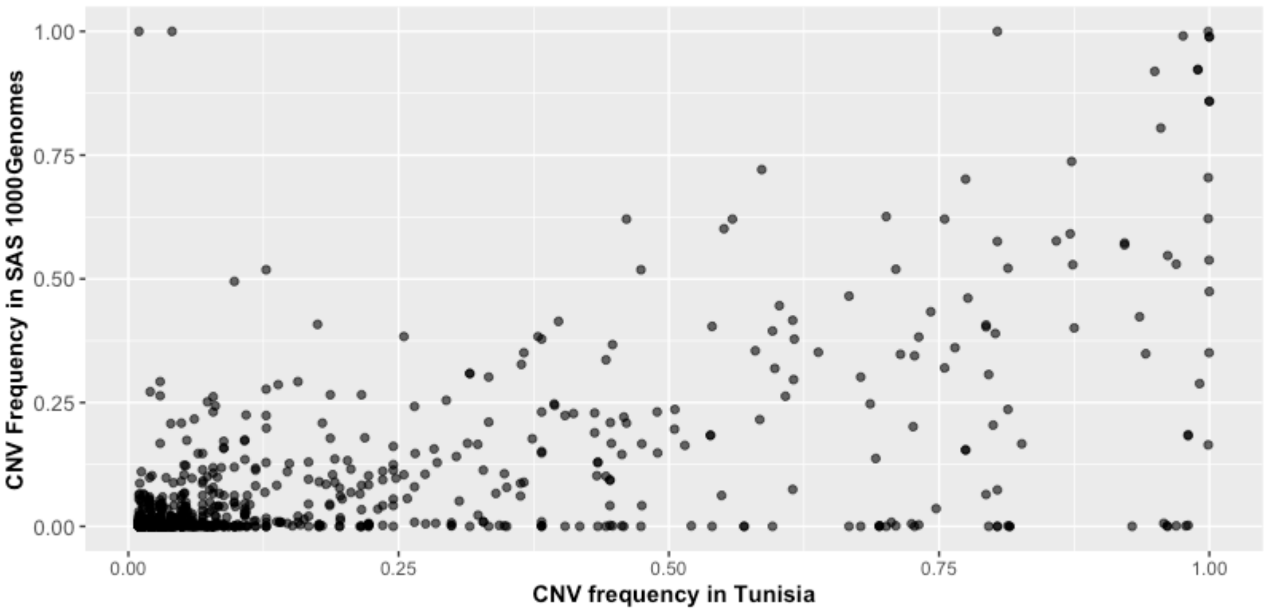

Figure 9e: Pairwise correlation between CNV frequency in Tunisia and SAS (1000Genomes)

Supplementary Figure 10: Maximum r^2 of bi-allelic deletions versus number of nearby SNPs in 200-kb windows

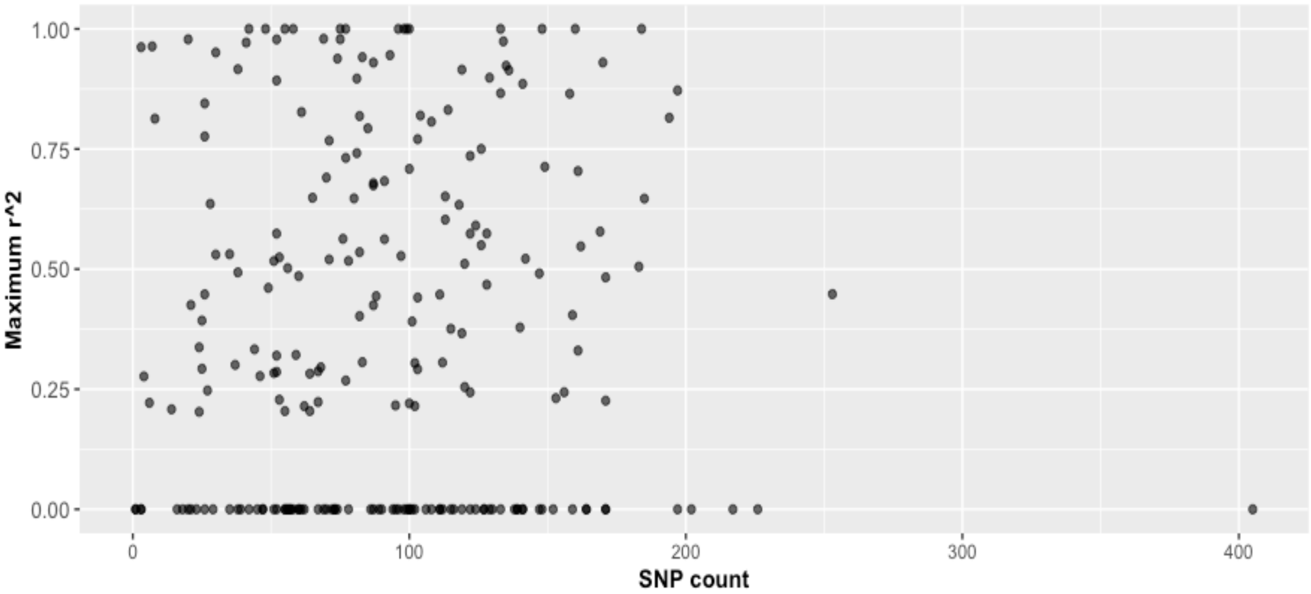

Figure 10: Maximum r^2 of bi-allelic deletions versus number of nearby SNPs in 200-kb windows

Supplementary Figure 11: Number of SNPs in strong correlation with the size of bi-allelic deletions

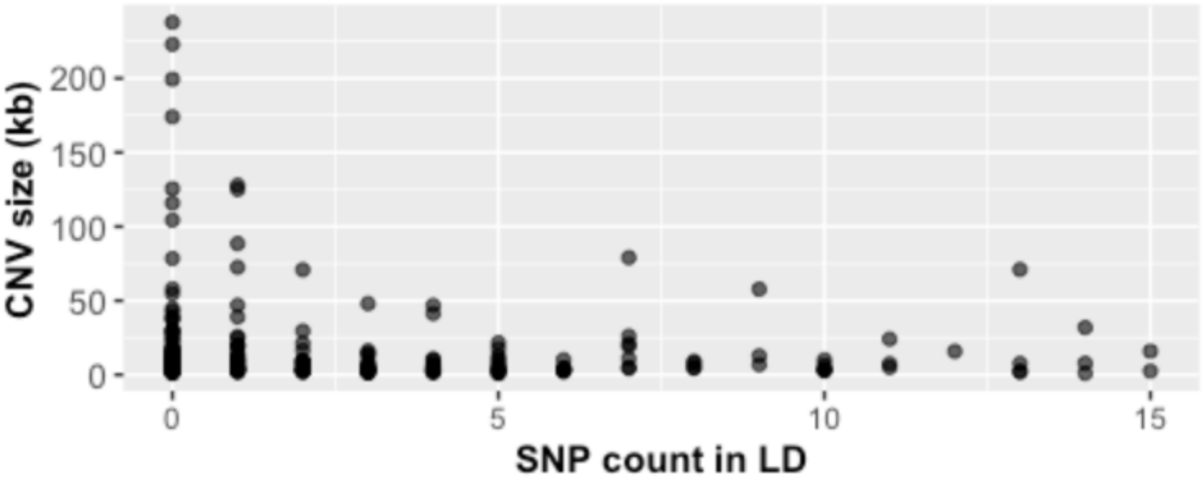

Figure 11: Number of SNPs in strong correlation with the size of bi-allelic deletions

Supplementary Table 1: Summary statistics of CNVs detected by PennCNV and BirdSuite

Summary statistics of CNVs	PennCNV	Birdsuite
CNV count	3964	6263
CNV carrier	102/135	102/135
Average number of CNVs/individual	38.86	61.4
Range of number of CNVs/individual	17 - 63	38-325
Median number of CNVs	39	60
Average size of CNVs (kb)	96.700	46.770
Median size of CNVs (kb)	28.420	18.120
Size range of CNVs	1.02 (kb) – 2.384 (Mb)	1.019 (kb) – 18.280 (Mb)
Proportion of deletions	2640 (66.6%)	4670
Average size (kb)	65.980	35.480
Median size (kb)	21.070	12.94
Size range	1.02 (kb) – 2.195(Mb)	1.019 (kb) – 1.787 (Mb)
Proportion of duplications	1324	1593
Average size (kb)	173	79.870
Median size (kb)	57.170	42.770
Size range	1.03(kb) - 2.384Mb	1.029 (kb)- 18.280(Mb)

Supplementary Table 2: Summary statistics of CNV regions (CNVR) detected by PennCNV and Birdsuite

**Method**	**PennCNV**	**Birdsuite**
**Total number of CNVRs identified**	751	1236
Mean size CNVRs identified (kb)	104	53.080
Median size of CNVRs identified (kb)	28.010	16.100
Genome coverage by CNVRs	78.072 (Mb)	65.607 (Mb)
Range	1.02 (kb) – 3.184 (Mb)	1.019 (kb) – 18.720 (Mb)
**Type of CNVR: Loss**		
Total number of deletions	469 (62.4%)	546 (44.2%)
Mean size CNVRs identified (kb)	49.260	34.750
Median size of CNVRs identified (kb)	**19.380**	13.330
Range	1.02(kb) – 2.074(Mb)	1.019 (kb) – 1.872 (Mb)
Genome coverage by CNVRs	23.102 (Mb)	18.971 (Mb)
**Type of CNVR: Gain**		
Total number of Gain	173 (23%)	603 (48.8%)
Mean size CNVRs identified (kb)	129.8	48.210
Median size of CNVRs identified (kb)	**61.430**	**16.140**
Genome coverage by CNVRs	22.458(Mb)	29.068 (Mb)
Range	2.570 (kb) – 1.828 (Mb)	1.031 (kb)- 1.828 (Mb)
**Type of CNVR: Mixed**		
Total number of mixed	109 (14.5 %)	87 (7 %)
Mean size CNVRs identified (Kb)	298.3	201.9
Median size of CNVRs identified (kb)	102.200	109.300
Range (kb)	1.4(kb) – 3.184 (Mb)	1.198 (kb) – 1.706 (Mb)
Genome coverage by CNVRs	32.510 (Mb)	17.566 (Mb)

Supplementary Table 3: CNVR size distribution

CNVR	PennCNV	Birdsuite
**Total**		
**<20 kb**	288 (0.3834)	697 (0.5639)
**>=20-<40 kb**	161 (0.21438)	204 (0.1650)
**>=40-<100 kb**	141 (0.1877)	182 (0.1472)
**>=100-<500 kb**	132 (0.1757)	140 (0.1132)
**>=500 kb**	29 (0.0386)	13 (0.0105)
Deletions		
**<20 kb**	243 (0.3235)	346 (0.2799)
**>=20-<40 kb**	99 (0.1318)	91 (0.0736)
**>=40-<100 kb**	87 (0.1158)	69 (0.0558)
**>=100-<500 kb**	35 (0.0466)	38 (0.0307)
**>=500 kb**	5 (0.0066)	2 (0.0016)
Duplications		
**<20 kb**	26 (0.0346)	337 (0.2726)
**>=20-<40 kb**	42 (0.0559)	101 (0.0817)
**>=40-<100 kb**	39 (0.0519)	96 (0.0776)
**>=100-<500 kb**	60 (0.0798)	65 (0.0525)
**>=500 kb**	6 (0.0079)	4 (0.0032)

Supplementary Table 4: Summary statistics of validated CNVRs (vCNVR)

Summary statistics of CNVRs	Validated CNVRs
**Total number of CNVRs identified**	586
Mean size CNVRs identified (kb)	90.300
Median size of CNVRs identified (kb)	28.290
Genome coverage by CNVRs (Mb)	52.913919
Range	1.020 kb – 2.074 (Mb)
**Type of CNVR: Loss**	
Total number of LOSSES	**327**
Mean size CNVRs identified (kb)	45.220
Median size of CNVRs identified (kb)	17.750
Range	1.020 kb - 2.074 (Mb)
Genome coverage by CNVRs	14.788 (Mb)
**Type of CNVR: Gain**	
Total number of Gain	**136**
Mean size CNVRs identified (kb)	125.900
Median size of CNVRs identified (kb)	55.080
Genome coverage by CNVRs	17.118 (Mb)
Range	3.433 (kb) – 1.828 (Mb)
**Type of CNVR: Mixed**	
Total number of mixed	**123**
Mean size CNVRs identified (Kb)	170.785
Median size of CNVRs identified (kb)	61.432
Range (kb)	1.4 (kb)- 1.797(Mb)
Genome coverage by CNVRs	21.006 (Mb)

Supplementary Table 5: vCNVR and CNP frequency summary

	CNV Type	CN=0	CN=1	CN=3	CN=4	CN=5	CN=6
Minimum frequency (%)	**vCNVR**	0	0	0	0	NA	NA
	CNP	0.98	0.98	0.98	0.98	2.17	1.12
Maximum frequency (%)	**vCNVR**	37.25	47.05	45.1	23.53	NA	NA
	CNP	99	73.96	71.15	92.39	71.43	20

Legend: NA; Not available

Supplementary Table 6: Summary statistics of CNP

**Summary statistics of CNPs**	**CNP**
**Total number of CNP genotypes**	**683**
Mean size CNP identified (kb)	22.827
Median size of CNP identified (kb)	7.050
Genome coverage by CNP (Mb)	15.591
Range (kb)	1.017 – 487.878
**Type of CNVR: Loss**	
Total number of Loss	**417**
Mean size CNP identified (kb)	15.342
Median size of CNP identified (kb)	5.937
Range	1.017- 222.892
Genome coverage by CNP (Mb)	6.397
**Type of CNP: Gain**	
Total number of Gain	**113**
Mean size CNP identified (kb)	41.489
Median size of CNP identified (kb)	14.024
Genome coverage by CNP (Mb)	4.688
Range (kb)	1.585 - 409.886
**Type of CNP: Mixed**	
Total number of mixed	**153**
Mean size CNP identified (Kb)	29.446
Median size of CNP identified (kb)	7.242
Range (kb)	1.133 – 487.878
Genome coverage by CNP (Mb)	4.505

Supplementary Table 7: Summary statistics of CNV global map

**Summary statistics of CNVs**	**CNV**
**Total number of CNV identified**	**1083**
Mean size SV identified (kb)	56.734
Median size of SV identified (kb)	13.475
Genome coverage by SV (Mb)	61.443 Mb
Range	1.017 (kb) - 2.074 (Mb)
**Type of CNV: Loss**	
Total number of losses	**622**
Mean size SV identified (kb)	31.497
Median size of SV identified (kb)	10.084
Range	1.017 (kb) – 2.074 (Mb)
Genome coverage by SV	19.591 (Mb)
**Type of CNV: Gain**	
Total number of Gain	**236**
Mean size SV identified (kb)	90.715
Median size of SV identified (kb)	29.943
Genome coverage by SV	21.204 (Mb)
Range	1.585 (kb)- 1.828 (Mb)
**Type of CNV: Mixed**	
Total number of mixed	**225**
Mean size SV identified (Kb)	91.118
Median size of SV identified (kb)	12.577
Range (kb)	1.399 (kb)- 1.797 (Mb)
Genome coverage by SV	20.250 (Mb)

Supplementary Table 8: CNV list spanning miRNA genes

CNV ID	chrom:start-end	CNV Length (kb)	CNV TYPE	Novel ?	microRNA gene name	Frequency in Tunisia (%)
CNVR_1_4	1:16813920-17262247	448.327	MIX	No	MIR3675	36.27
CNVR_1_5	1:17594895-17620009	25.114	DUP	No	MIR3972	0.98
CNVR_1_27	1:146101239-147929336	1828.097	DUP	No	MIR5087; MIR6077	0.98
CNVR_11_21	11:81653513-81681585	28.072	DUP	No	MIR4300HG	0.98
CNVR_14_11	14:50399836-50519022	119.186	DUP	Yes	MIR6076	0.98
CNVR_15_9_CNP2082	15:34700683-34862723	162.040	MIX	No	MIR1233-1; MIR1233-2	27.45
CNVR_15_18	15:76868226-76895775	27.549	MIX	No	MIR3713	45.10
CNVR_16_3_CNP2141	16:14989863-15116257	126.394	MIX	No	MIR1972-1; MIR1972-2; MIR3179-1; MIR3179-2; MIR3179-3; MIR3179-4; MIR3180-1; MIR3180-2; MIR3180-3; MIR3670-1; MIR3670-2; MIR3670-3; MIR3670-4; MIR6511A1; MIR6511A2; MIR6511A3; MIR6511A4; MIR6770-1; MIR6770-2; MIR6770-3	55.88
CNVR_16_5	16:18286514-18783174	496.660	DUP	No	MIR3179-1; MIR3179-2; MIR3179-3; MIR3179-4; MIR3180-1; MIR3180-2; MIR3180-3; MIR3670-1; MIR3670-2; MIR3670-3; MIR3670-4; MIR6511A1; MIR6511A2; MIR6511A3; MIR6511A4; MIR6770-1; MIR6770-2; MIR6770-3	2.94
CNVR_16_15	16:70024588-70213044	188.456	MIX	No	MIR1972-1; MIR1972-2	3.92
CNVR_16_18	16:76884555-77939493	1054.938	MIX	No	MIR4719	1.96

CNVR_17_4	17:14094261-15491545	1397.284	MIX	No	MIR4731	2.94
CNVR_2_21	2:87250495-88052330	801.835	MIX	No	MIR4435-1; MIR4435-2; MIR4771-1; MIR4771-2	7.84
CNP10324	2:87887217-87971885	84.668	MIX	No	MIR4435-1; MIR4435-2	3.23
CNVR_2_25	2:112050882-112196233	145.351	DUP	No	MIR4435-1; MIR4435-2; MIR4435-2HG	0.98
CNVR_21_3	21:19950029-20416913	466.884	MIX	Yes	MIR548X; MIR548XHG	1.96
CNVR_22_5_CNP2563	22:25663985-25994339	330.354	MIX	No	MIR6817	9.80
CNVR_3_44	3:195383141-195482370	99.229	MIX	No	MIR570; MIR570HG	25.49
CNVR_4_5	4:21450943-21467397	16.454	DEL	No	MIR7978	0.98
CNVR_4_8	4:38841353-39120619	279.266	DUP	Yes	MIR574	0.98
CNVR_7_20	7:71964741-72325006	360.265	DUP	No	MIR4650-1; MIR4650-2	1.96

Supplementary Table 9: GO annotation of genes affected by CNVs in the Tunisian population

Category	GO Term	Gene Count	Fold Enrichment	p-value
Biological Process	detection of chemical stimulus involved in sensory perception of smell	38	3.26	5.28 x10-10
Biological Process	negative regulation of cell differentiation	11	7.87	9.88 x10-7
Biological Process	G-protein coupled receptor signaling pathway	49	1.99	6.92 x10-6
Biological Process	homophilic cell adhesion via plasma membrane adhesion molecules	16	3.70	2.92 x10-5
Biological Process	detection of chemical stimulus involved in sensory perception	12	4.61	5.58 x10-5
Biological Process	negative regulation of growth	6	11.53	1.26 x10-4
Biological Process	female pregnancy	11	4.51	1.56 x10-4
Biological Process	glutathione derivative biosynthetic process	6	9.95	2.67 x10-4
Biological Process	glutathione metabolic process	8	5.21	7.94 x10-4
Biological Process	cellular response to zinc ion	5	9.61	0.0015
Biological Process	cell adhesion	25	1.99	0.0019
Biological Process	nitrobenzene metabolic process	3	27.38	0.0043
Biological Process	drug metabolic process	5	6.76	0.0059
Biological Process	steroid metabolic process	6	5.09	0.0061
Biological Process	regulation of alpha-amino-3-hydroxy-5-methyl-4-isoxazole propionate selective glutamate receptor activity	4	9.17	0.0087
Biological Process	epoxygenase P450 pathway	4	8.11	0.0122
Biological Process	nervous system development	16	2.03	0.0128
Biological Process	xenobiotic catabolic process	3	15.64	0.0143
Biological Process	flavonoid biosynthetic process	4	7.30	0.0164
Biological Process	flavonoid glucuronidation	4	6.64	0.0212
Biological Process	carbohydrate metabolic process	11	2.31	0.0215
Biological Process	receptor localization to synapse	3	12.17	0.0236
Biological Process	presynaptic membrane assembly	3	12.17	0.0236
Biological Process	lung epithelial cell differentiation	3	10.95	0.0290
Biological Process	sensory perception of smell	10	2.29	0.0308
Biological Process	cardiac conduction	5	4.06	0.0340
Biological Process	auditory receptor cell stereocilium organization	3	9.95	0.0348
Biological Process	lymphocyte chemotaxis	4	5.21	0.0401
Biological Process	metabolic process	10	2.17	0.0415

Biological Process	ventricular septum morphogenesis	4	5.03	0.0438
Biological Process	protein localization to synapse	3	8.42	0.0476
Cellular Component	integral component of membrane	194	1.31	1.01 x10-5
Cellular Component	cell junction	28	2.12	3.65 x10-4
Cellular Component	postsynaptic membrane	15	2.47	0.0032
Cellular Component	organelle membrane	9	3.59	0.0035
Cellular Component	Golgi apparatus	39	1.57	0.0060
Cellular Component	integral component of plasma membrane	57	1.40	0.0088
Cellular Component	synapse	12	2.30	0.0156
Cellular Component	plasma membrane	140	1.18	0.0174
Molecular Function	olfactory receptor activity	38	3.39	1.8339 x10-10
Molecular Function	G-protein coupled receptor activity	46	2.49	3.03 x10-8
Molecular Function	transmembrane signaling receptor activity	22	3.95	1.90 x10-7
Molecular Function	alpha-amylase activity	5	38.45	2.19 x10-6
Molecular Function	glutathione transferase activity	8	8.79	2.70 x10-5
Molecular Function	IgG binding	5	17.48	1.28 x10-4
Molecular Function	carbohydrate binding	14	2.75	0.0019
Molecular Function	thiolester hydrolase activity	3	16.48	0.0129
Molecular Function	carboxylic ester hydrolase activity	5	4.81	0.0196
Molecular Function	voltage-gated calcium channel activity	5	4.81	0.0196
Molecular Function	CCR chemokine receptor binding	4	6.69	0.0209
Molecular Function	glutathione binding	3	10.49	0.0316
Molecular Function	guanyl-nucleotide exchange factor activity	8	2.61	0.0341
Molecular Function	cation binding	3	9.61	0.0373
Molecular	glucuronosyltransferase activity	4	5.30	0.0385

Function				
Molecular Function	transferase activity	7	2.80	0.0388

Supplementary Table 10: Disease class of genic CNVs

GAD disease class	Number of genes	Fold Enrichment	p-value
Chemdependency	169	1.73	2.94x10-16
Hematological	77	1.88	4.5x10-8
Cardiovascular	159	1.39	4.62x10-7
Metabolic	184	1.30	2.36x10-6
Immune	112	1.4	6.58x10-5
Psych	78	1.47	3.07x10-4
Unknown	63	1.45	1.9x10-3
Neurological	101	1.30	2.34x10-3
Vision	28	1.51	3x10-2
Aging	36	1.41	3.27x10-2
