# Supplementary material for: A map of copy number variations in the Tunisian population: a valuable tool for medical genomics in North Africa
Source: NPJ Genom Med. 2021 Jan 8;6:3. doi: 10.1038/s41525-020-00166-5 (PMC7794582; doi:10.1038/s41525-020-00166-5)
Supplement: Supplementary file 3 — Reporting Summary [file 41525_2020_166_MOESM3_ESM.pdf]

nature research

Corresponding author(s): Romdhane Lilia

Last updated by author(s): Oct 24, 2020

Reporting Summary

Nature Research wishes to improve the reproducibility of the work that we publish. This form provides structure for consistency and transparency in reporting. For further information on Nature Research policies, see our Editorial Policies and the Editorial Policy Checklist.

Statistics

For all statistical analyses, confirm that the following items are present in the figure legend, table legend, main text, or Methods section.

n/a	Confirmed	
☐	☒	The exact sample size (*n*) for each experimental group/condition, given as a discrete number and unit of measurement
☒	☐	A statement on whether measurements were taken from distinct samples or whether the same sample was measured repeatedly
☐	☒	The statistical test(s) used AND whether they are one- or two-sided<br>*Only common tests should be described solely by name; describe more complex techniques in the Methods section.*
☐	☒	A description of all covariates tested
☒	☐	A description of any assumptions or corrections, such as tests of normality and adjustment for multiple comparisons
☐	☒	A full description of the statistical parameters including central tendency (e.g. means) or other basic estimates (e.g. regression coefficient) AND variation (e.g. standard deviation) or associated estimates of uncertainty (e.g. confidence intervals)
☐	☒	For null hypothesis testing, the test statistic (e.g. *F*, *t*, *r*) with confidence intervals, effect sizes, degrees of freedom and *P* value noted<br>*Give P values as exact values whenever suitable.*
☒	☐	For Bayesian analysis, information on the choice of priors and Markov chain Monte Carlo settings
☒	☐	For hierarchical and complex designs, identification of the appropriate level for tests and full reporting of outcomes
☐	☒	Estimates of effect sizes (e.g. Cohen's *d*, Pearson's *r*), indicating how they were calculated

Our web collection on statistics for biologists contains articles on many of the points above.

Software and code

Policy information about availability of computer code

Data collection	No custom code was used. Open source softwares have been used

Data analysis

* Affymetrix Power Tool (APT) v1.8.6 was used to obtain genotype calls required for copy number estimation
* PLINK v1.07 (Purcell et al., 2007): SNP quality control and linkage desequilibrium analysis
* PennCNV v.1.0.3 (Wang et al., 2007): CNV calling
* Birdsuite v1.5.5 (Korn et al., 2008): CNV calling
* BedTools v2.25.0 (Quinlan et al, 2010): CNV quality control processing and analysis
* DAVID (Huang et al., 2009): CNV functionnal annotation
* Database of Genomic Variants (DGV) (http://dgv.tcag.ca/dgv/app/home): CNV annotation
* dbVar (https://www.ncbi.nlm.nih.gov/dbvar/): CNV annotation
* AnnotSV v2.3 (Geoffroy et al., 2018): Structural and functional annotation
* 1000Genomes project database (Sudmant et al., 2015): CNV annotation
* gnomAD database (Collins et al., 2019): CNV annotation
* RegulomeDB database (http://www.regulomedb.org) (Boyle et al., 2012): SNP functional annotation
* Ensembl (https://www.ensembl.org): SNP functional annotation
* ClinVar (https://www.ncbi.nlm.nih.gov/clinvar/): SNP functional annotation
* NHGRI-GWAS catalog (https://www.ebi.ac.uk/gwas/) : SNP functional annotation
* The statistical software R version 3.6.2 (http://www.r-project.org): statistical analysis
* R package ggplot2 (Wickham, 2016): Data visualization
* R package RCircos (Zhang et al., 2013): CNV genome-wide distribution ploting

For manuscripts utilizing custom algorithms or software that are central to the research but not yet described in published literature, software must be made available to editors and reviewers. We strongly encourage code deposition in a community repository (e.g. GitHub). See the Nature Research guidelines for submitting code & software for further information.

Data

Policy information about availability of data

All manuscripts must include a data availability statement. This statement should provide the following information, where applicable:

- Accession codes, unique identifiers, or web links for publicly available datasets
- A list of figures that have associated raw data
- A description of any restrictions on data availability

Datasets supporting the conclusions of this article are included within the article, supplementary tables and supplementary data. Other data that support the findings of this study are available from the corresponding author on reasonable request. In Tunisia, genetic data are considered as personal private data, for these reasons we have submitted the minimal dataset as supporting files but we are not allowed to submit the full raw data. The full raw data may be made available upon request by other investigators and after approval of our IRB

Field-specific reporting

Please select the one below that is the best fit for your research. If you are not sure, read the appropriate sections before making your selection.

☒ Life sciences ☐ Behavioural & social sciences ☐ Ecological, evolutionary & environmental sciences

For a reference copy of the document with all sections, see nature.com/documents/nr-reporting-summary-flat.pdf

Life sciences study design

All studies must disclose on these points even when the disclosure is negative.

Sample size	135 healthy (free from any genomic disorder) unrelated Tunisian individuals. After QC, 102 individuals were kept for subsequent analysis
Data exclusions	*Sixteen individuals were excluded from subsequent analysis as they had QC contrast values greater than 0.4.<br>*Individuals with poor quality of signal intensity were removed (17 individuals) if they had more than 100 CNV segments detected, along with wave factor > 0.05 or Log2R standard deviation > 0.4 and B-allele drift > 0.0125.
Replication	Not applicable
Randomization	Not applicable
Blinding	Not applicable

Reporting for specific materials, systems and methods

We require information from authors about some types of materials, experimental systems and methods used in many studies. Here, indicate whether each material, system or method listed is relevant to your study. If you are not sure if a list item applies to your research, read the appropriate section before selecting a response.

Materials & experimental systems

n/a	Involved in the study	
☒	☐	Antibodies
☒	☐	Eukaryotic cell lines
☒	☐	Palaeontology and archaeology
☒	☐	Animals and other organisms
☐	☒	Human research participants
☒	☐	Clinical data
☒	☐	Dual use research of concern

Methods

n/a	Involved in the study	
☒	☐	ChIP-seq
☒	☐	Flow cytometry
☒	☐	MRI-based neuroimaging

Human research participants

Policy information about studies involving human research participants

Population characteristics	A total of 135 healthy (free from any genomic disorder) unrelated Tunisian individuals (103 males and 32 females) originating from Northern, Central and Southern Tunisia has been recruited. Participants' mean age is 48+/- 10 years.
Recruitment	135 healthy (free from any genomic disorder) unrelated Tunisian individuals have been recruited
Ethics oversight	The biomedical ethics committee of Pasteur Institute of Tunis

Note that full information on the approval of the study protocol must also be provided in the manuscript.
